# Laser reprogramming magnetic anisotropy in soft composites for reconfigurable 3D shaping

**DOI:** 10.1038/s41467-020-20229-6

**Published:** 2020-12-10

**Authors:** Heng Deng, Kianoosh Sattari, Yunchao Xie, Ping Liao, Zheng Yan, Jian Lin

**Affiliations:** 1 Department of Mechanical and Aerospace Engineering, University of Missouri, Columbia, MO 65211 USA; 2 Department of Biomedical, Biological, and Chemical Engineering, University of Missouri, Columbia, MO 65211 USA; 3 Department of Electrical Engineering and Computer Science, University of Missouri, Columbia, MO 65211 USA; 4 Department of Physics and Astronomy, University of Missouri, Columbia, MO 65211 USA

**Keywords:** Polymers, Actuators, Polymers

## Abstract

Responsive soft materials capable of exhibiting various three-dimensional (3D) shapes under the same stimulus are desirable for promising applications including adaptive and reconfigurable soft robots. Here, we report a laser rewritable magnetic composite film, whose responsive shape-morphing behaviors induced by a magnetic field can be digitally and repeatedly reprogrammed by a facile method of direct laser writing. The composite film is made from an elastomer and magnetic particles encapsulated by a phase change polymer. Once the phase change polymer is temporarily melted by transient laser heating, the orientation of the magnetic particles can be re-aligned upon change of a programming magnetic field. By the digital laser writing on selective areas, magnetic anisotropies can be encoded in the composite film and then reprogrammed by repeating the same procedure, thus leading to multimodal 3D shaping under the same actuation magnetic field. Furthermore, we demonstrated their functional applications in assembling multistate 3D structures driven by the magnetic force-induced buckling, fabricating multistate electrical switches for electronics, and constructing reconfigurable magnetic soft robots with locomotion modes of peristalsis, crawling, and rolling.

## Abstract (web summary)

Responsive soft materials which can exhibit various three-dimensional (3D) shapes under the same stimulus are desirable for applications in adaptive and reconfigurable soft robots. Here, the authors report a laser rewritable magnetic composite film, whose responsive shape-morphing behaviors induced by a magnetic field can be digitally and repeatedly reprogrammed by a facile method of direct laser writing.

## Introduction

Inspired by adaptive shape changes of natural species, responsive soft materials that are capable of complex, reversible, and fast shape transformations upon external stimuli have attracted great interests in recent decades^1–5^. These materials can be fabricated from hydrogel, shape memory polymers (SMPs), and their composites with various types of additives. They can respond to stimuli including moisture, organic solvents, light, heat, magnetic field, and electric field. They have demonstrated potential applications in emerging fields such as metamateriallis, soft robotics, sensors, and multifunctional scaffolds^6–12^. The responsive behaviors of these materials are determined by spatially designated anisotropies by the ways such as differential crosslinking degrees^13,14^ and multilayered structures^15–17^. Recently, our group reported that the spatial anisotropies of polymeric crystal phases^18^ and swellable guest medium in organogel^19^ can also induce the responsive shape changes. However, these programmed anisotropies are permanently determined, causing them to display only a single and predetermined shape transformation mode under a certain stimulus^19–22^. Fabrication of responsive soft materials that show reprogrammable shape transformation modes under the same stimulus is still a grand challenge^23^, while it is highly demanded for broadening their applications in fields such as adaptive and reconfigurable soft robots.

A magnetic soft composite composed of magnetic particles embedded in a polymer matrix shows such a possibility, although little related work has been demonstrated. The magnetic particles enable to generate a magnetic anisotropy by creating a nonuniform magnetization profile^24–26^. The anisotropy generates magnetic forces and torques in different directions in response to an actuation magnetic field, driving the preprogrammed 3D shape transformation of the magnetic soft composite. These instantaneous and spatiotemporal responses to the magnetic stimulus—that can be remotely actuated^24,27,28^—make them the promising materials for application in soft robots. To encode the magnetic anisotropy, there are two common strategies: making magnetization after fabrication and making magnetic reorientation during the fabrication. In the first strategy, the magnetic particles are first fixed to form a soft composite. The magnetic soft composite is then deformed (folded, bended, or wrapped) followed by magnetization under a strong magnetic field (>1 T)^21,29–31^. The resulting material enables simple magnetic deformation modes, such as folding and bending for applications in locomotion robots^30^ or soft grippers^31^. This process leads to a magnetic anisotropy that cannot be precisely and digitally controlled, thus possessing a limited shape-programming freedom.

In the second strategy, the magnetic anisotropy can be programmed during the fabrication processes such as photolithography and 3D printing, in which the magnetic particles are reoriented in polymer precursor solution under a programming magnetic field. As the solution can be selectively cured, a localized magnetization profile can be patterned^24–26^. This strategy possesses a higher programming freedom, thus leading to more complex shape transformation modes. For instance, the resulting materials enable complex robotic locomotion^26^ or auxetic behaviors^24^. Nevertheless, once the magnetization profile (or called magnetic anisotropy) is patterned and fixed, they cannot be reconfigured. Therefore, these magnetic soft materials exhibit a single shape transformation mode under the same actuation magnetic field. Even though the induced magnetic force and torque can be tuned by changing the magnetic direction and/or flux of the actuation magnetic field^28^, their shape transformation modes have not been maximized due to the intrinsic restriction of the fixed magnetic anisotropy in the fabricated materials. Thus, in order to obtain numerous reconfigurable shape changing modes in a single magnetic soft material, the one whose magnetic anisotropy can be repeatedly and digitally programmed is highly desired.

In this work, we report a magnetic responsive soft material (MRSM), whose magnetic anisotropy can be digitally and repeatedly reprogrammed by a facile method of direct laser writing (DLW). We demonstrated multiple shape transformation modes in a single MRSM film under the same actuation magnetic field. To apply this material, we first demonstrated assembly of responsive reconfigurable 3D architectures by the magnetic actuation of the developed MRSM substrates. Moreover, this concept was utilized to fabricate multifunctional and shape-reconfigurable devices such as multistate electrical switches for electronics. Finally, the magnetic soft robots that can show various locomotion such as peristalsis, crawling, and rolling were reported. This responsive soft material would pave a way to fabricating adaptive, reconfigurable magnetic soft microrobots, minimally invasive medical implantation devices, flexible electronics, and active mechanical metamaterials.

## Results

**Fig. 1:**
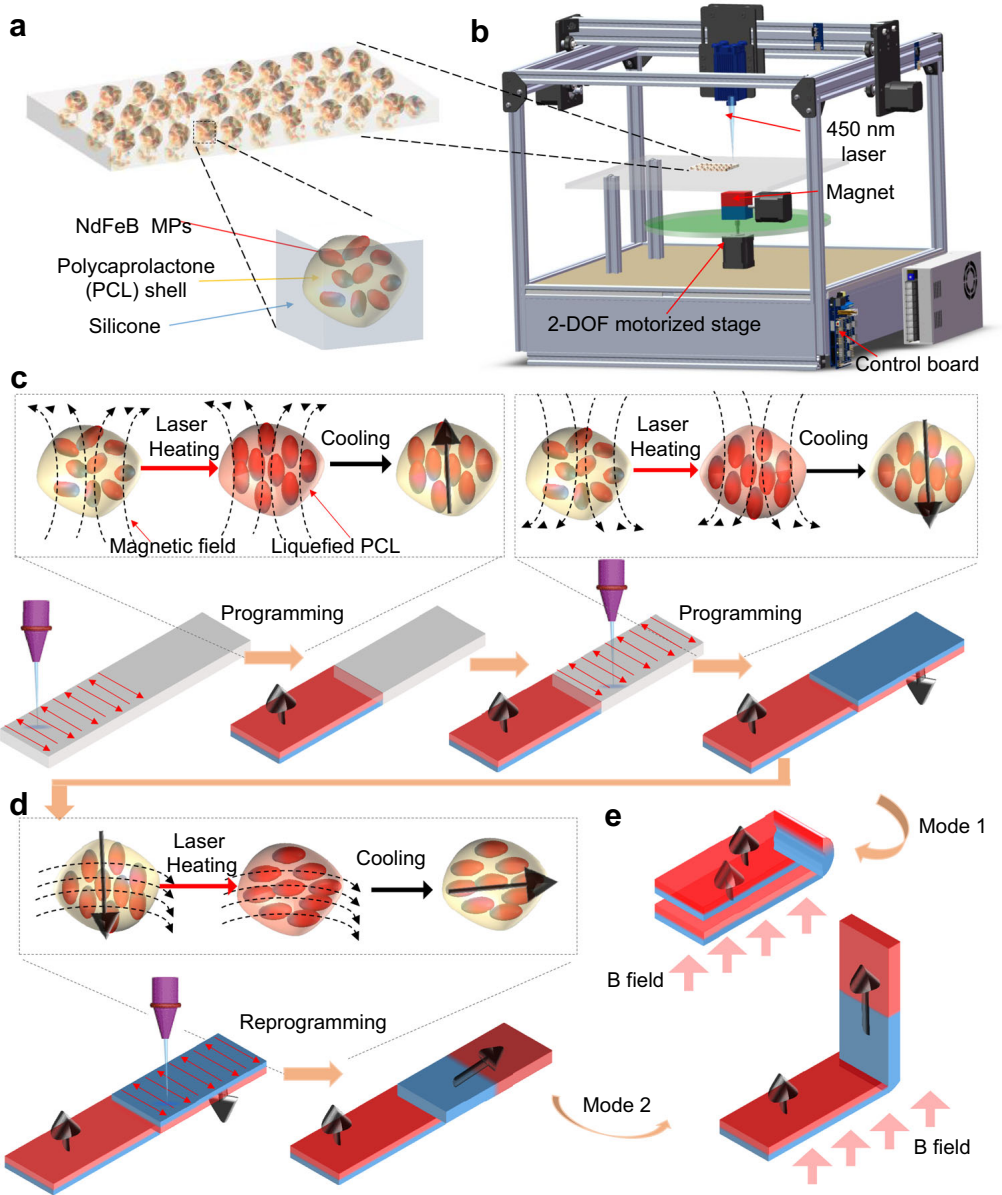
System design and mechanism of reprogramming the magnetic anisotropy in MRSM films by DLW. **a** Schematic showing the structure of a MRSM film, which consists of silicone and magnetic NdFeB MPs wrapped by PCL shells. **b** Schematic of a customized DLW system for programming a magnetic anisotropy in a MRSM film. **c** Schematic showing steps of programming a magnetic anisotropy in a MRSM film. **d** Schematic showing steps of reprogramming a magnetic anisotropy in the same MRSM film to the one shown in **c**. **e** Schematic showing mechanism of multiple shape transformation modes in a reprogrammable MRSM film.

In the work, we used a phase change polymer, such as Polycaprolactone (PCL), to encapsulate NdFeB microparticles (MPs) to form the PCL encapsulated NdFeB MPs (NdFeB@PCL MPs) (Supplementary Fig. 1a). These resulting NdFeB@PCL MPs were then mixed with the silicone matrix to fabricate the MRSM films. The process is illustrated in Fig. 1a with details shown in Supplementary Fig. 1b and in “Methods” section. In MRSM, the NdFeB MPs also function as the photothermal agents, which absorb photo energy from the laser irradiation and then convert it to heat to melt the PCL shells. A customized DLW system to achieve such a goal is shown in Fig. 1b. Details of the system design and construction are described in Supplementary Fig. 2. To align the MRSM film to the laser scanning pathway, laser induced graphene^32^ was formed on the polyimide surface as a marker (Supplementary Fig. 2b), which enables to position registration without visual access to the laser. Such position registration by using LIG as the marker guarantees the accuracy of the laser programming. The patterning resolution is estimated to be ~300 μm, rendering a soft robot in a millimeter scale (Supplementary Fig. 3). The control algorithm for the system is illustrated in Supplementary Fig. 4. The reason of using the DLW technique is that the laser generates a transient heat on the exposed regions^32^. The transient heat can effectively limit the thermal diffusion to the non-exposed regions, thus resulting in a good patterning resolution. In addition, rapid cooling enables quick fixation of the magnetization patterns in the exposed regions, thus avoiding interference when the direction of the programming magnetic field is changed during the continuous laser writing. Finally, DLW can be digitally controlled to generate various patterns in a high-precision manner^32–34^.

The working mechanism for reprogramming the magnetic anisotropy is described as follows. Initially, the NdFeB@PCL MPs are randomly oriented in an as-prepared MRSM film, resulting in no total magnetization. As the PCL shell melts due to the localized laser heating, the magnetic orientations of the encapsulated NdFeB MPs are realigned to the direction of the programming magnetic field (Fig. 1c). The reason of choosing PCL is that its melting temperature is much lower than the silicone, so that the laser power can be controlled to minimize damage to the silicone. After the laser is removed from the exposed areas, PCL is quickly solidified, thus fixing the programmed magnetic orientations of the NdFeB MPs in the patterned domains. By digitally controlling the laser scanning meanwhile dynamically changing the directions of the programming magnetic field, the magnetic domains with different magnetic orientations can be realized for a complex magnetic anisotropy in a MRSM film (Fig. 1c). As the phase change of PCL is reversible, the programmed magnetic anisotropy can be easily rewritten into a different one by following the same procedure (Fig. 1d). By this way, diversified shape transformation modes can be reconfigured for various magnetic actuations in the same MRSM film (Fig. 1e).

**Fig. 2:**
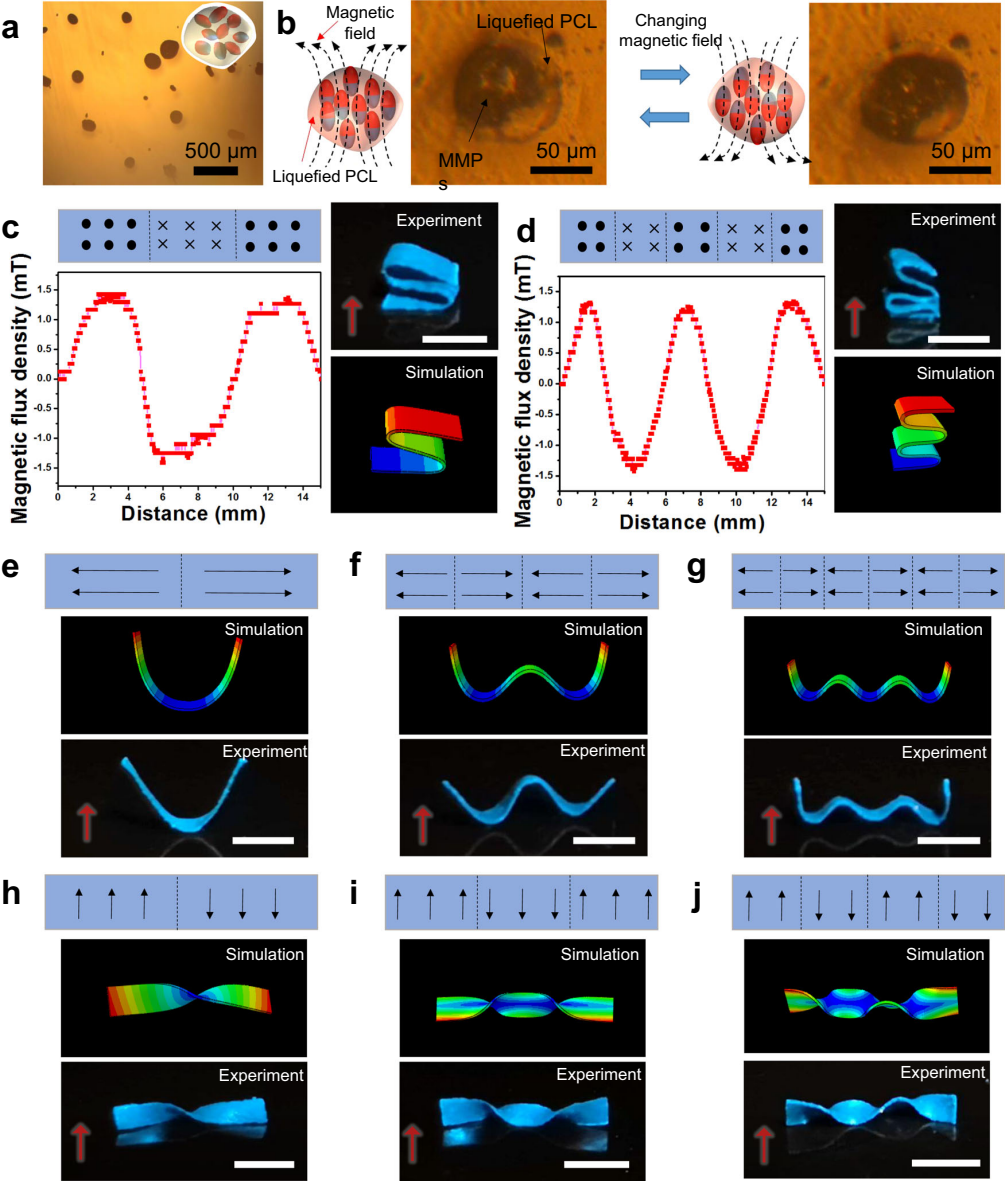
Mechanism study on programming and reprogramming magnetic anisotropies in MRSM films. **a** An optical image of NdFeB@PCL MPs. **b** Schematic and optical images showing reorientation of NdFeB MPs in NdFeB@PCL MPs by an external magnetic field when heated above the melting temperature of PCL. **c**, **d** Schematic of two different magnetization patterns in a MRSM strip, their corresponding magnetic flux density distributions, morphed 3D shapes for the actuated MRSM strip under the same actuation magnetic field and the expected 3D shapes resulting from FEA simulation. **e**–**j** Schematic of laser programmed magnetization patterns, the corresponding 3D shapes simulated by FEA, and really actuated 3D shapes in the same MRSM strip. The strip is actuated under a magnetic field of 150 mT, which is perpendicular to the strip as indicated by the red arrow. Scale Bars: 5 mm.

The NdFeB MPs have an average size of 5 μm (Supplementary Fig. 5), which were pre-magnetized in a 1.1 T uniform magnetic field formed by two N52 1-inch permanent magnets with a 3-mm apart^26^. After they are mixed with PCL, the obtained NdFeB@PCL MPs, each of which contains multiple NdFeB MPs, have enlarged sizes of ranging from 80 to 190 μm (Fig. 2a). If heated above the melting temperature of PCL (>60 °C), the PCL shells of the NdFeB@PCL MPs melt, causing reorientation of the encapsulated NdFeB MPs by the programming magnetic field (Fig. 2b and Supplementary Movie 1). Besides acting as the magnetic agents in the MRSM, the NdFeB MPs can also function as the photothermal agents. Their light absorption capability was confirmed by the UV–vis–NIR measurement. As shown in Supplementary Fig. 6, the pure silicone and PCL films show almost no light absorption in the wavelength range of 400–900 nm, while the NdFeB@PCL and MRSM films show >98% light absorption in the same wavelength range. Fabrication of the MRSM films was further optimized by tuning the fabrication parameters, such as mass loading of NdFeB MPs in the NdFeB@PCL MPs, mass loading of the NdFeB@PCL MPs in the MRSM films, and laser power (Supplementary Figs. 7 and 8). Supplementary Fig. 7 shows that the optimum mass loading of NdFeB MPs in the NdFeB@PCL MPs is 50% in order to achieve a maximized magnetic flux density after the laser programming. By studying the effect of the laser power (Supplementary Fig. 8), we determined that the optimized laser power to achieve the maximized magnetic flux density in the MRSM was 0.06 W. To balance the magnetic and mechanical properties, the MRSM films with 50 wt% NdFeB@PCL MPs were chosen for the following experiments (Supplementary Fig. 8). The magnetization of the MRSM film reaches to ~1.2 mT, which is compared to the fabricated samples shown in a previous study^26^. The laser induced reorientation of NdFeB MPs yields a magnetic flux density of ~90% of the saturated magnetic flux density in the MRSM films, which is much higher than those realized by a 3D printing method^24^.

As an illustrative example to demonstrate the ability of digitally programming magnetic anisotropy, an alternating magnetization pattern with three discrete domains was realized in a MRSM strip (Fig. 2c, 15 mm × 2 mm × 0.2 mm). The magnetic flux densities of the domains were measured by a commercial magnetic sensor (Supplementary Fig. 9) to obtain a distribution curve (Fig. 2c). On the two adjacent domains, magnetic torques with opposite magnetic directions were generated under the same actuation magnetic field. Meanwhile, in this magnetization pattern, the boundaries across the adjacent patterns form the folding creases for an origami pattern. Along these folding creases, the strip folds into an “S” shape under a 150 mT actuation magnetic field, which is perpendicular to the strip as indicated by the red arrow (Fig. 2c). Upon removal of the actuation magnetic field, the deformed MRSM strip quickly recovers to its original flat shape (Supplementary Movie 2). Then the same MRSM strip was reprogrammed with a new alternated magnetization pattern having five discrete domains (Fig. 2d). The measured magnetic flux distribution of these domains is shown in Fig. 2d. A new zigzag shape was realized from this new magnetic anisotropy under the same actuation magnetic field (Fig. 2d). It should be noted that the leftmost segment of magnetic film was fixed to the ground by water washable glue to guarantee the stable shape transformations (Supplementary Fig. 10).

Due to the high design freedom of DLW, the discrete magnetic domains can be arbitrarily created to form distinguished magnetization patterns in the same MRSM film. To achieve desirable 3D shape morphing, finite element analysis (FEA) was used to design the magnetic anisotropy. A FEA model proposed by Zhao et al. was used to numerically simulate the deformation of the MRSM films in response to an actuation magnetic field^24^. In the simulation, the MRSM was considered as an incompressible material, in which magnetic moment (**M**) is exposed to an external magnetic field (**B**), generating a magnetic stress tensor of, where operator ⊗ represents a dyadic product and **F** is a deformation gradient tensor. To simulate the shape transformation, the magnetic stress tensor was introduced in a user-defined element subroutine in ABAQUS. The FEA simulation results were in good agreement with the experimental ones (Fig. 2c, d), suggesting that the FEA simulation is capable of guiding the design of magnetic shape-morphing structures driven by the programmed magnetic anisotropy. By using the FEA model-guided design, the same MRSM strip was reprogramed to have various pre-determined magnetic anisotropies for morphing of a wide range of 3D shapes (Fig. 2e–j). Under the same actuation magnetic field, the MRSM strip can be reshaped into wave-like shapes with 1–3 pits (Fig. 2e–g) and twisted shapes with 1–3 nodes (Fig. 2h–j), all of which are in good agreement with the FEA prediction. To investigate reversibility and stability of programming the magnetic anisotropies, a cycling test was conducted. The magnetic anisotropies were alternatively programmed in the same MRSM strip, and then the shape morphing was actuated for 100 cycles. During the test, the shape-morphing behaviors were well maintained (Supplementary Fig. 11), suggesting the excellent reprogramming ability of the MRSM strip.

**Fig. 3:**
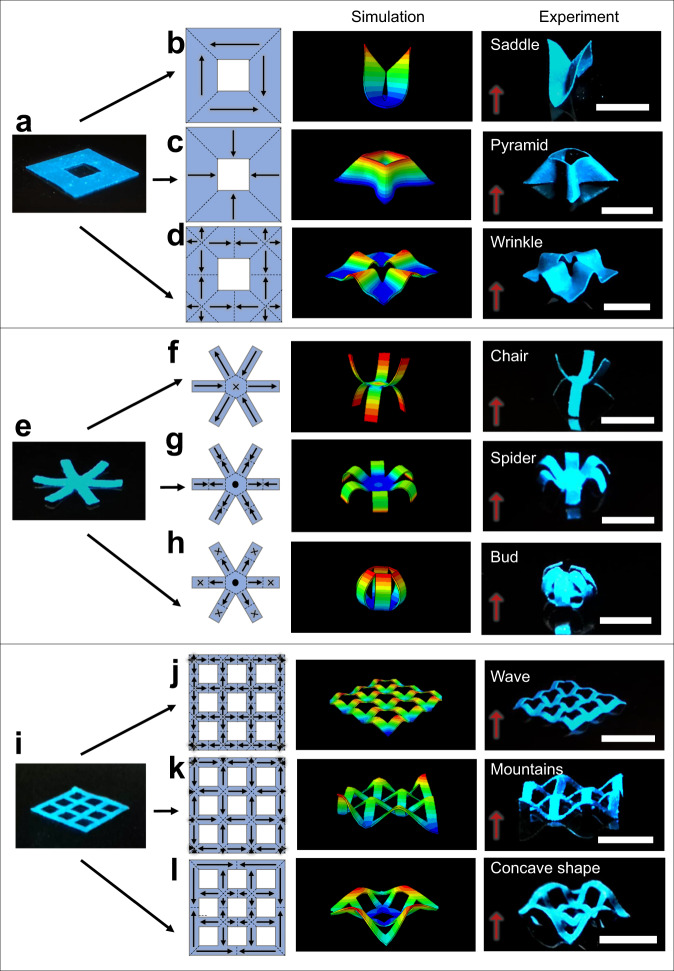
Demonstration of complex 3D shaping under magnetic actuation of MRSM origami films with reprogrammable magnetization patterns. Schematic of magnetization patterns, FEA predictions and optical images of morphed 3D structures from a square-hollow MRSM film (**a**–**d**), a six-armed MRSM film (**e**–**h**) and a mesh-shaped MRSM film (**i**–**l**). The magnetic films are actuated under a magnetic field of 150 mT, which is perpendicular to the films as indicated by the red arrow. Scale Bars: 5 mm.

More complex magnetic anisotropies can be programmed and then reprogramed in the same MRSM films. Using the origami principle, we designed three representative patterns of the magnetic anisotropies in a square-hollow MRSM film (Fig. 3a–d and Supplementary Movie 3), a six-armed MRSM film (Fig. 3e–h and Supplementary Movie 4), and a mesh-shaped MRSM film (Fig. 3i–l and Supplementary Movie 5), respectively. Under an actuation magnetic field of 150 mT that is perpendicular to their planes, these MRSM films are morphed to distinguished 3D shapes. In Fig. 3b, c, we designed the same magnetization patterns but with opposite magnetization direction. The FEA simulations predict that they should yield the saddle and pyramid shapes, which were later proved by the experiment. If the same film was programmed to have more origami magnetization patterns that vary in magnitude, it is expected to afford increased complexity in the morphed 3D geometries (Fig. 3d). To further illustrate this facile magnetic anisotropy programming method, we implemented other origami magnetization patterns with different angles of the magnetization directions in a six-armed MRSM film (Fig. 3e). Depending on the magnetization patterns the MRSM film can be transformed to chair, spider, and bud shapes, respectively (Fig. 3f–h). When more intricate design of the origami magnetization patterns were introduced to a mesh-shaped MRSM film (Fig. 3i), the 2D mesh is transformed to auxetic structures with distinguished 3D shapes. Both of the 3D shapes in Fig. 3j, k exhibit alternating and symmetric peaks and valleys, which are transformed from the equidistant magnetic domains although the distance in each domain is different. This shape symmetry can be broken by simply programming the magnetization domains by changing their distances to result in a 3D concave shape (Fig. 3l). This result was further proved by the FEA simulation. As remote actuation of untethered auxetic structures has shown a promise for application in active mechanical metamaterials^24^, the reconfigurable shape transformation enabled by the proposed laser rewriting of the MRSM films would pave a route to making such devices multifunctional. Moreover, the reprogrammability of the MRSM films will broaden the potential of responsive materials in various fields from both economic and environmental aspects.

**Fig. 4:**
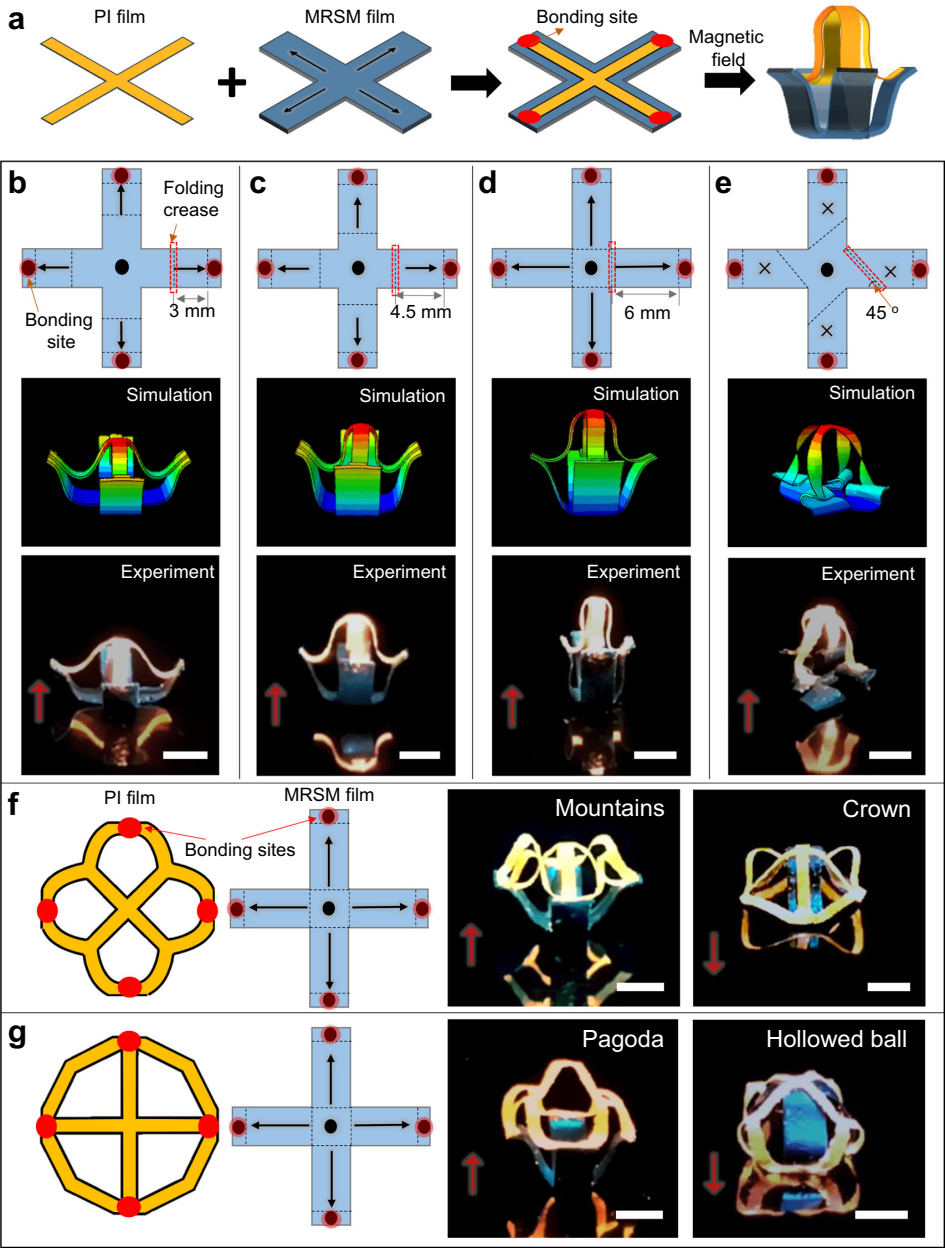
Functional demonstration of MRSM substrates for responsive 3D structure assembly. **a** Schematic showing fabrication of a buckled 3D structure driven by a magnetically responsive MRSM substrate. **b**–**e** Schematic of four types of magnetization patterns in a MRSM substrate, FEA prediction, and optical images of buckled 3D structures with different buckling heights (**b**–**d**) and a twisted 3D structure (**e**). The polyimide (PI) film was treated by fluorescent dye to facilitate the observation. **f**, **g** Two representative PI patterns, schematic of magnetization patterns of a MRSM substrate, and optical images of buckled 3D structures. Red dots indicate the bonding sites between PI films and MRSM substrates. Red arrows indicate the directions of the actuation magnetic field of 150 mT. Scale Bars: 5 mm.

MRSM with reprogrammable magnetic anisotropies have a wide spectrum of applications. First, they are compatible with the recently demonstrated responsive buckling assembly for fabrication of 3D architectures^35–37^. To fabricate them, passive 2D films are selectively bonded onto the responsive substrates. Upon external stimulation, responsive substrates start to morph their shapes and then impart compressive force to the 2D passive films via the bonding sites, thus controllably buckling the 2D passive films to well-defined 3D architectures. However, in these studies, the buckling behaviors are fixed and non-reconfigurable due to the programmed permanent anisotropies in their responsive substrates. Herein, we demonstrated a reconfigurable and responsive mechanical buckling assembly that was actuated by the developed MRSM substrate. The fabrication steps are shown in Fig. 4a and Supplementary Fig. 12. First, a polyimide (PI) pattern is selectively bonded onto a MRSM substrate with a geometry of a cross. The MRSM substrate is programmed with a specific magnetization pattern. Upon the magnetic actuation, the morphing of the MRSM substrate buckles the PI pattern into a 3D shape. According to previous literatures^12,38–42^, the buckling behaviors of the passive layer are quite influenced by the relative distances of the bonding sites on the elastomer substrates. Compared with the existing techniques, our method shows that such distance can be controlled by the folding creases, which can be easily modulated by programming different magnetic anisotropies in the MRSM substrate. Herein, the distances of the cases shown in Fig. 4b–d are 3.0 mm, 4.5 mm, and 6.0 mm, respectively. Under a perpendicular magnetic field of 150 mT, the same MRSM substrate can buckle the same PI pattern into 3D dome-shapes with different heights (Fig. 4b–d and Supplementary Movie 6), which agree well with the FEA simulation results. If the magnetization pattern was programmed in such a way that the folding creases are not perpendicular to the longitudinal axil of the arms, e.g., a 45° angle, the bonding sites would be deviated from the axil of the arm, thereby deforming the PI pattern to a twisted 3D dome shape (Fig. 4e and Supplementary Movie 6). More complicated 3D architectures can be achieved by increasing the geometric complexity of the passive PI films. As shown in Fig. 4f, g, two types of kirigami PI patterns are selectively bonded to a cross-shaped MRSM substrate with the same magnetization pattern to the one shown in Fig. 4d. Once exposed to a perpendicular magnetic field, the MRSM substrate buckles the two PI kirigami patterns into mountains-shaped and pagoda-shaped 3D architectures, respectively (Fig. 4f, g). If the actuation magnetic field is switched in the opposite direction, the two kirigami PI patterns are transformed into the 3D crown and ball shapes, respectively (Fig. 4f, g).

**Fig. 5:**
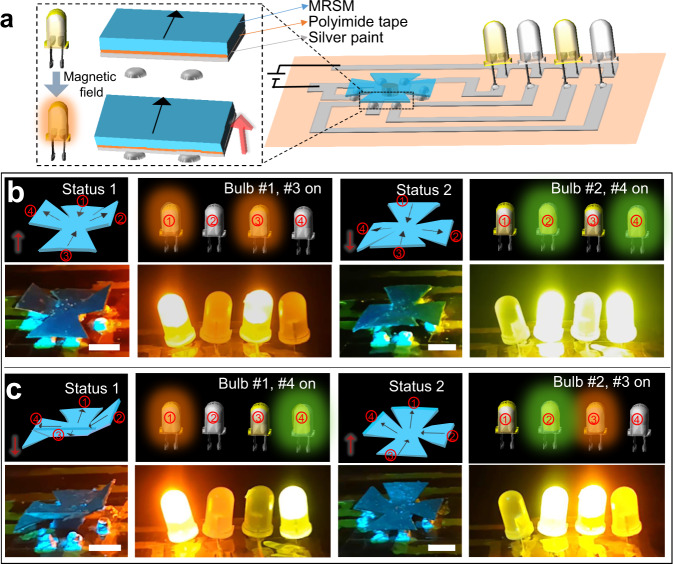
Functional demonstration of MRSM as a multistate electrical switch. **a** Schematic of a circuit made of a reconfigurable MRSM switch to control the LEDs. **b**, **c** experimental results showing multistate switching of the MRSM switch. The magnetic switch is actuated by a magnetic field of 150 mT, which is perpendicular to the device as indicated by the red arrow. Scale Bars: 5 mm.

In addition, the reprogrammable MRSM film can also be used as a multistate electrical switch for electronics, e.g., light-emitting diodes (LEDs). To demonstrate that, we fabricated a four-petal MRSM film attached a PI film which was coated by silver paint. The function of the PI is to provide enough mechanical support to make the MRSM switch be suspended above the electrodes (Fig. 5a). Initially, the circuit that connects four LEDs is open. By using the DLW technique, a magnetization pattern as shown in Fig. 5b is programmed. With such a magnetization pattern, the Petal 1 and Petal 3 bend to connect partial circuit to turn on LED 1 and LED 3 upon the actuation of a perpendicular magnetic field (150 mT). Once the actuation magnetic field is applied in the opposite direction, Petal 2 and Petal 4 connect the electrodes and switch on LED 2 and LED 4 (Supplementary Movie 7). After the magnetic anisotropy in the petals of this MRSM switch is reprogrammed, e.g., making the opposite magnetization directions of Petal 1 and Petal 2, the switch can yield a different switching function. By subsequently changing the magnetic directions of the actuation magnetic field, LED 1/LED 4 and LED 2/LED 3 can be subsequently turned on (Fig. 5c).

**Fig. 6:**
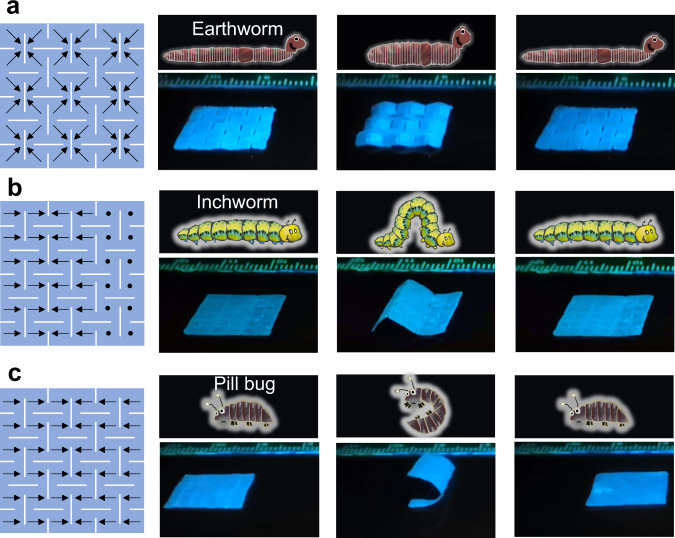
Locomotion modes of soft robots that are fabricated from the MRSM kirigami film but with different laser written magnetization patterns. The soft robots can mimic the peristalsis motion of an earthworm (**a**), crawling motion of an inchworm (**b**), and rolling motion of a pill bug (**c**).

Finally, we explored the application of MRSM in the reconfigurable magnetic soft robots. We fabricated a MRSM kirigami film which was subsequently programmed to possess three different types of magnetization patterns (Fig. 6). Upon application of a moving magnet (Supplementary Fig. 13), three distinguished locomotion modes that mimic the motions of livings such as peristalsis of an earthworm, crawling of an inchworm, and rolling of a pill bug, were achieved. To mimic the peristalsis motion of an earthworm, which moves by alternately contracting and extending the body, a periodical magnetization pattern is programmed in the MRSM kirigami film (Fig. 6a). When applying the magnetic field, the MRSM soft robot contracts and then quickly recovers to its original shape when the magnetic field fades as magnet passes the soft robot (Supplementary Fig. 14). This process drastically releases the elastic and magnetic potential energy, thus making the soft robot move forward. By repeating the actuation process, the MRSM soft robot repeats this locomotion mode like the peristalsis of an earthworm, which moves 0.7 cm after ten cycles of motion. (Fig. 6a, Supplementary Fig. 15 and Supplementary Movie 8). To mimic the crawling of an inchworm, which folds its body into an arch structure during the movement, an alternated magnetization pattern is encoded in the MRSM film as shown in Fig. 6b. Upon the magnetic actuation, the MRSM soft robot folds into an arch structure, and then quickly recovers to its flat state when the magnetic field fades as magnet passes the soft robot (Supplementary Fig. 16). By repeating the actuation process, the MRSM soft robot moves forward by cycling movement of arching and stretching just like a creeping inchworm, which moves 3.2 cm after ten cycles of motion. (Fig. 6b, Supplementary Fig. 17 and Supplementary Movie 9). If a symmetric magnetization pattern is reprogrammed in the same MRSM film (Fig. 6c), by using the same actuation process (Supplementary Fig. 18), we can expect a rolling locomotion of the MRSM soft robot as a pill bug does, which moves 4.6 cm after three cycles of motion. (Fig. 6c, Supplementary Fig. 19 and Supplementary Movie 10).

## Discussion

In summary, we developed a novel MRSM with shape-reconfiguration capability. By a home-made magnetic laser writing system, the arbitrary magnetic patterns can be programmed in the MRSM films. Upon magnetic actuation, the encoded magnetic anisotropies rapidly deform the MRSM films into complex 3D structures. Because the solid–liquid phase change of PCL is reversible, the programmed magnetic anisotropies can be reprogrammed to different ones. Therefore, multiple reversible shape transformation modes were realized in the same MRSM film by the same actuation magnetic field. Finally, functional demonstrations of the MRSM films in the magnetic responsive assembly of the 3D architectures, the multistate electrical switches for electronics, and the reconfigurable soft robots were made. The study would pave a route to constructing multifunctional and responsive devices and systems. Moreover, the proposed method is facile and can be extended to other soft composites by varying phase changing materials or elastomer matrixes. As the lasers have various wavelengths and their patterning resolution can be down to sub-micrometer, fabrication of microscale soft robots can be expected.

## Methods

### Fabrication of MRSM films

NdFeB MPs were first magnetized in a 1.1 T magnetic field which was formed by two N52 1-inch permanent magnets with a distance of 3 mm. The magnetized NdFeB MPs were then mixed with liquid PCL on an 80 °C hotplate to obtain an NdFeB@PCL composite. After that, 0.4 g NdFeB@PCL composite was mixed homogeneously with 10 ml polyvinyl alcohol (PVA) aqueous solution (20 wt%) on a 100 °C hotplate. Due to the phase separation, NdFeB@PCL MPs were formed and dispersed in the PVA solution. Then, the NdFeB@PCL MPs were purified by a centrifuge in water at 1000 rpm for three times. To fabricate the elastomer matrix, part A and part B of commercial silicone solution (Ecoflex 00-30) were mixed at a volume ratio of 1:1 at room temperature. Then the resulting solution was mixed with the NdFeB@PCL MPs. Finally, the mixture was poured into a mold and cured to the MRSM films. The obtained MRSM films were cut into various geometries by a CO_2_ laser. The magnetic patterns in the MRSM films were programmed by our home-made laser writing system with an in situ controlled magnetic field (Supplementary Fig. S2). The patterns were designed in an open source software Inkscape (https://inkscape.org/) and then formatted to G-code by the extension of Inkscape. The G-code controls the movement of the laser beams and the rotation of the programming magnet through an algorithm as illustrated in Supplementary Fig. S4.

### Material characterizations

Optical images of NdFeB MPs, NdFeB@PCL MPs, and MRSM were taken using an optical microscope (AmScope). UV–Vis–NIR absorption spectra of a PCL film, a silicone elastomer film, an NdFeB@PCL film, a MRSM film were measured by a Perkin Elmer Lambda 35 UV−Vis spectrometer. Tensile testing of MRSM was conducted on a Mark-10 ESM303 tensile tester. The videos and photographs of shape-morphing behaviors of MRSM were taken by a smart phone under a blue LED filtered by a polyimide tape.

### FEA simulations

In all the simulations, the deformed structures in response to the actuation magnetic fields were simulated by a user-defined element (UEL) subroutine. It was proposed by Zhao et al.^24^ and modified in this study, implemented in the commercial finite-element analysis software ABAQUS. The following input parameters were used for all the simulations: Young’s modulus of *E* = 1 MPa and an actuation uniform magnetic field of 150 mT. The magnetic flux density of 1.2 mT was used for MRSM films.

## Supplementary information

Supplementary Information

Peer Review File

Description of Additional Supplementary Files

Supplementary Movie 1

Supplementary Movie 2

Supplementary Movie 3

Supplementary Movie 4

Supplementary Movie 5

Supplementary Movie 6

Supplementary Movie 7

Supplementary Movie 8

Supplementary Movie 9

Supplementary Movie 10

## Data Availability

The data that support the findings of this study are available from the corresponding authors upon reasonable request. See Author Contributions for the responsible persons for specific data sets.

## Code availability

The code used for FEA simulations is available at https://github.com/linresearchgroup/Laser-Reprogramming-Magnetic-Anisotropy.
