# Supplementary material for: Laser reprogramming magnetic anisotropy in soft composites for reconfigurable 3D shaping
Source: Nat Commun. 2020 Dec 10;11:6325. doi: 10.1038/s41467-020-20229-6 (PMC7730436; doi:10.1038/s41467-020-20229-6)
Supplement: Supplementary file 1 — Supplementary Information [file 41467_2020_20229_MOESM1_ESM.pdf]

Supplementary Information for

Laser Reprogramming Magnetic Anisotropy in Soft Composites for Reconfigurable 3D Shaping

Heng Deng[1#], Kianoosh Sattari[1#], Yunchao Xie[1], Ping Liao[1], Zheng Yan[2], Jian Lin[1,3,4]*

[1]Department of Mechanical and Aerospace Engineering
[2]Department of Biomedical, Biological, and Chemical Engineering
[3]Department of Electrical Engineering and Computer Science
[4]Department of Physics and Astronomy
University of Missouri, Columbia, Missouri 65211, USA.
[#]Authors equally contributed to the work.
[*]Email: linjian@missouri.edu

Supplementary Figures

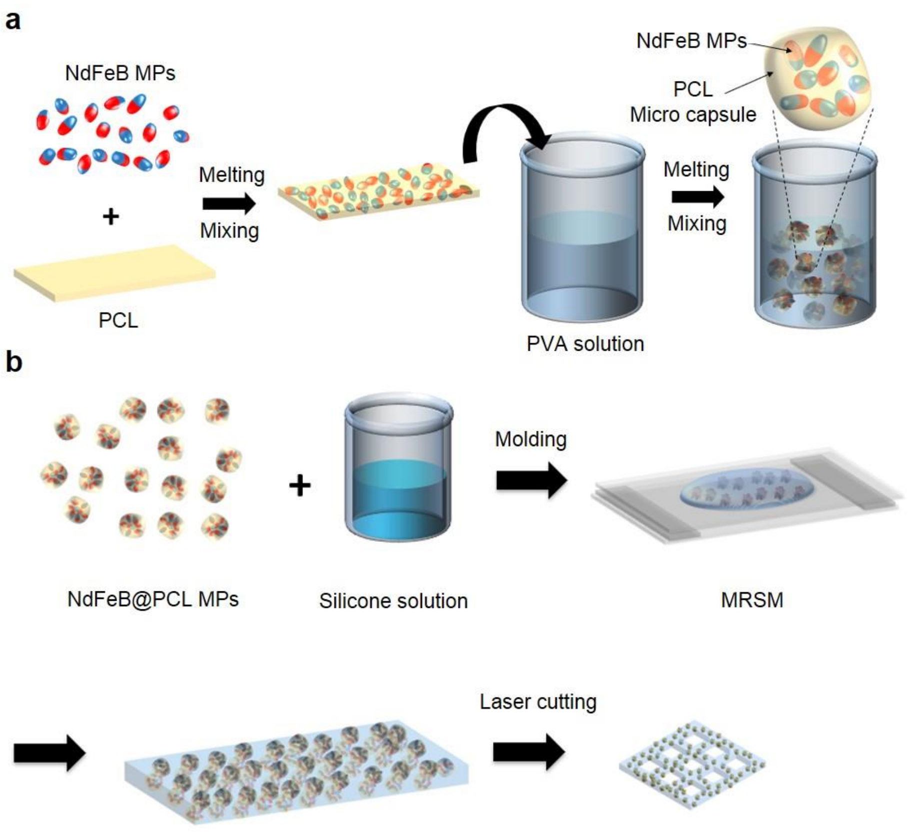

Supplementary Fig. 1. Fabrication steps of a magnetic responsive soft material (MRSM).

a, Magnetized NdFeB MPs were mixed with the liquid PCL on an 80 ℃ hotplate to obtain an NdFeB@PCL composite. Then, the PCL@NdFeB composite was mixed homogeneously with

polyvinyl alcohol (PVA) aqueous solution on a 100 °C hotplate. Due to the phase separation, NdFeB@PCL MPs were formed and dispersed in the PVA solution. **b,** The obtained NdFeB@PCL MPs were mixed with silicone precursor solution and then poured into a mold. After cured, the resulted MRSM was cut into a desired shape by a CO_2 laser.

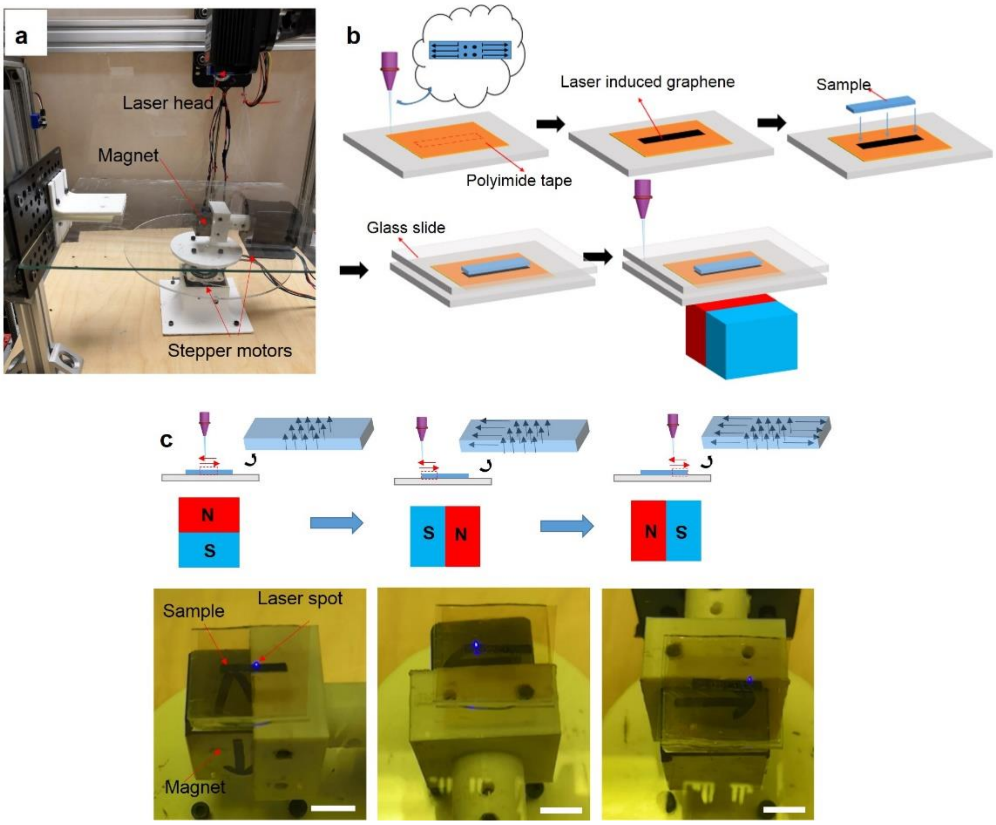

Supplementary Fig. 2. Programming of the magnetic anisotropy in MRSM film by DLW. **a,** Photograph of a customized direct laser writing (DLW) system. **b,** Schematic showing the process of position registration. A polyimide (PI) tape is placed on the DLW platform. A polyimide (PI) tape was placed on the DLW platform, then a blue laser (0.1 W) scanned the PI surface to induce laser induced graphene (LIG). As the laser scanning pathway is the same to the one used for programming the magnetic anisotropy, the LIG can be used as the marker for position registration. Finally, a glass slide is covered on the sample to fix the position of sample.

After that, the sample is ready for laser programming. **c,** Photographs showing the process of programming the magnetic anisotropy in a MRSM film by DLW and varying the direction of a programming magnetic field. The DLW system consists of a 450 nm laser and a 2-DOF motorized cube permanent magnet. They were controlled by an Adurino board for automating the programming process. The Adurino board controls the pathway of a laser head (speed = 200 mm/min) and also the direction of the magnetic field from a cube permanent magnet. Upon the laser irradiation, the photothermal heat absorbed by the NdFeB MPs locally melts the PCL shell of the NdFeB@PCL MPs. As a result, the encapsulated NdFeB MPs are reoriented along the directions of the applied magnetic field. By controlling the rotation of the cube permanent magnet, arbitrary 3D magnetization patterns can be programmed in the laser-exposed regions. Upon cooling, PCL is solidified to fix the orientation of the NdFeB MPs to freeze the written magnetization patterns. Such a magnetic programming procedure can be repeated to reconfigure the magnetic anisotropies in the same MRSM film.

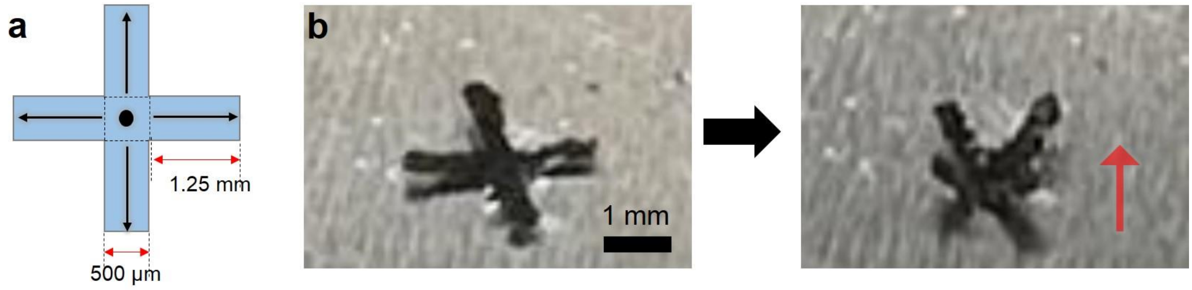

Supplementary Fig. 3. Demonstration of a millimeter-scale magnetic soft robot. a, Schematic of a millimeter-scale four-armed soft robot. The size of each arm is 1.25 mm x 500 μm. **b,** Shape transformation of a four-armed soft robot under an actuation magnetic field of 150 mT, which is perpendicular to the films as indicated by the red arrow.

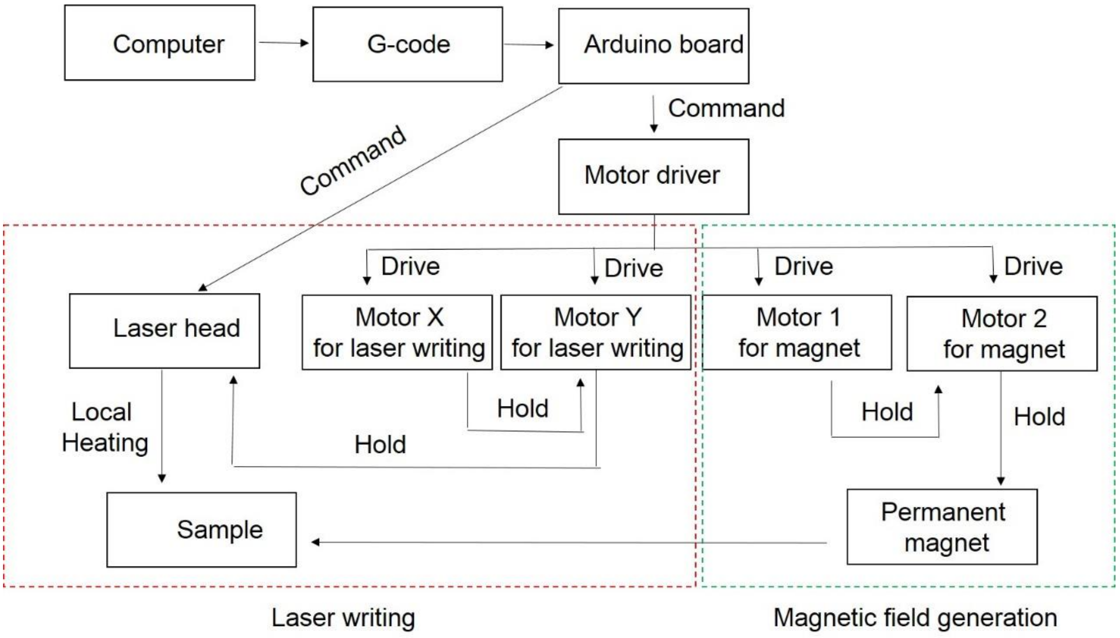

Supplementary Fig. 4. Control algorithm for the laser writing system. Schematic showing the signal flow and hardware control of a DLW system for programming the magnetic anisotropies in the MRSM films.

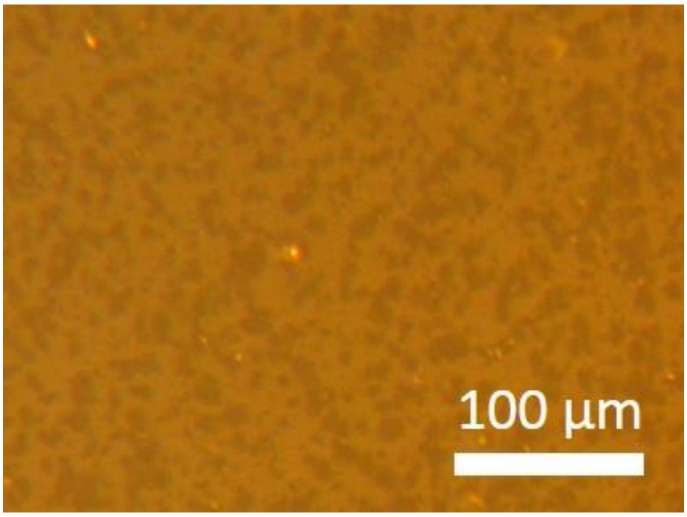

Supplementary Fig. 5. Optical image of NdFeB microparticles. The NdFeB MPs have an average size of 5 µm.

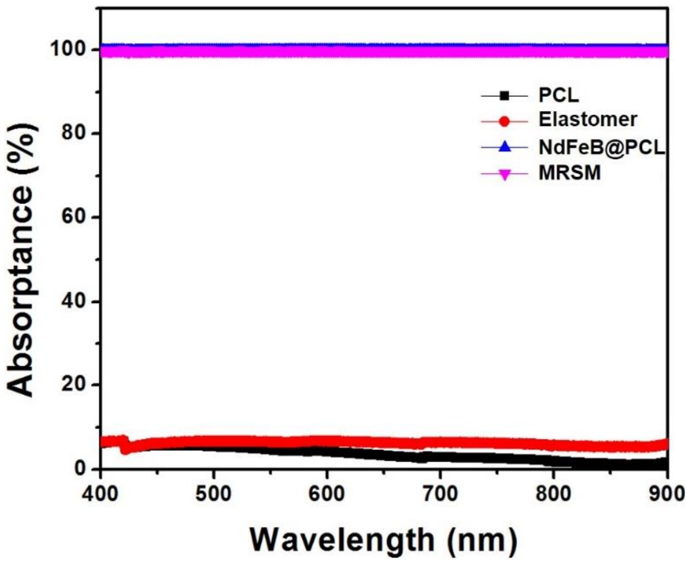

Supplementary Fig. 6. Light absorption capability of the MRSM film. UV-vis-NIR absorption spectra of a PCL film, a silicone elastomer film, an NdFeB@ PCL composite film, a MRSM film.

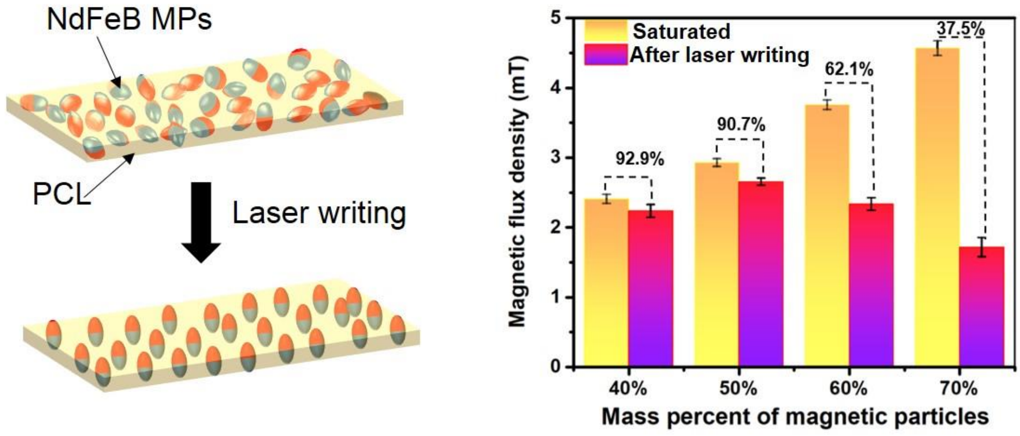

Supplementary Fig. 7. Magnetic flux densities of the NdFeB@PCL composite with different mass loading of NdFeB MPs. Error bars represent the standard deviation of the mean

Note: The NdFeB@PCL composite films (5 mm x 5 mm x 0.2 mm) with different mass loading of the NdFeB MPs are fabricated and programmed by the laser writing under a magnetic field of 180 mT and a laser power of 0.06 W. The measured magnetic flux density increases from

2.2 mT to 2.5 mT as the mass loading of the NdFeB MPs increases from 40% to 50%. However, when the mass percentage of the NdFeB MPs further increases, the measured magnetic flux density decreases. This is because high concentration of the NdFeB MPs may make the melted magnetic slurry highly viscous, resulting in difficulty of reorienting the NdFeB MPs in the composite. To better program the magnetic anisotropy, the optimum mass percentage of the NdFeB MPs in the NdFeB@PCL composite was determined to be 50%. We would use this optimized formula to prepare the NdFeB@PCL MPs. In addition, we fabricated NdFeB@PCL composite films with the non-magnetized NdFeB MPs and then magnetized them under an impulse field (about 1.1 T) to yield a magnetic flux density as high as possible (saturated magnetic flux density). In comparison, the programmed NdFeB@PCL composite produced by the proposed DLW method yields a magnetic flux density of ~ 90% of the saturated magnetic flux density in the MRSM with the same mass loading of NdFeB MPs.

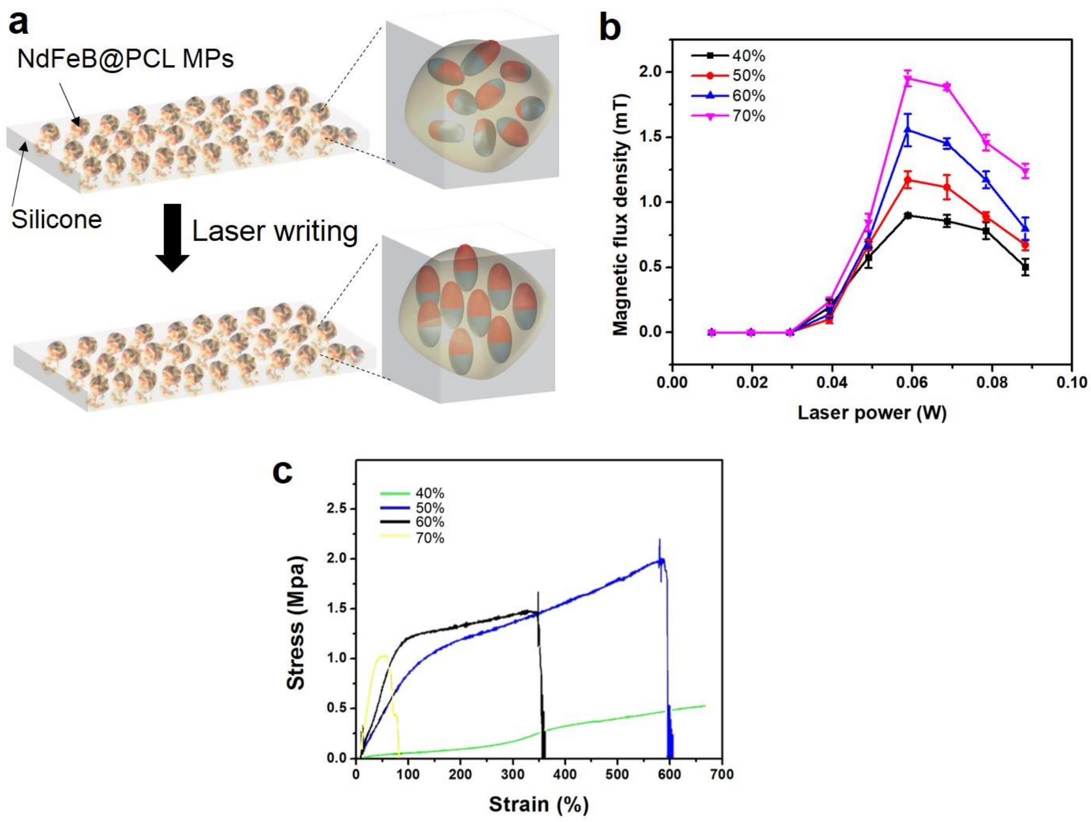

Supplementary Fig. 8. Magnetic and mechanical properties of the MRSM with different mass loading of NdFeB@PCL composite. a, Schematic showing steps of programming an magnetic anisotropy in a MRSM film. **b,** Effect of the laser power on the magnetic flux density of the MRSM films prepared with the varied mass loading of the NdFeB@PCL MPs. **c,** Tensile test of MRSM films with different mass loading of the NdFeB@PCL MPs. Error bars represent the standard deviation of the mean.

Note: NdFeB@PCL MPs with different mass loading were embedded into the silicone to fabricate a series of the MRSM films with dimensions of 5 mm x 5 mm x 0.2 mm. Then these films were programmed under a fixed magnetic field of 180 mT but varied laser powers from 0.01 W to 0.09 W (Supplementary Fig. 7a). As shown in Supplementary Fig. 7b, when the laser power is fixed, the measured magnetic flux density increases as the mass loading of the NdFeB@PCL MPs increases. However, the increased mass loading of the NdFeB@PCL MPs

decreases the softness and stretchability of the MRSM films (Supplementary Fig. 7c). To balance the magnetic and mechanical properties, the MRSM films with 50 wt% NdFeB@PCL MPs were chosen for the following experiments. Supplementary Fig. 7b shows that the magnetic flux density of MRSM is also influenced by the laser power. It increases when the laser power increases from 0.01 W to 0.06 W, but decreases when the laser power is larger than 0.06 W. We hypothesize that the laser power of < 0.06 W is not high enough to fully liquefy the PCL, resulting in partial alignment of the NdFeB MPs, while laser power of > 0.06 W may demagnetize the NdFeB MPs due to the increased temperature. The optimized laser power for programing was 0.06 W and used for the following experiments.

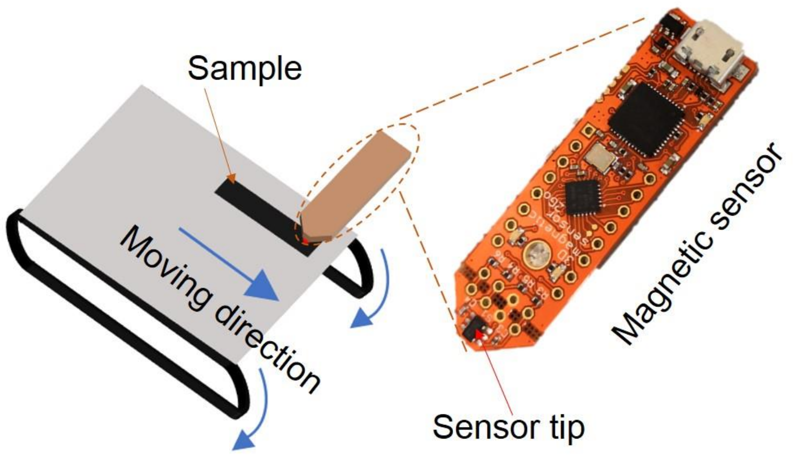

Supplementary Fig. 9. Schematic showing measurement of the magnetic flux density distribution in a MRSM strip. During the measurement, the MRSM strip was fixed on a moving stage. A commercial magnetic sensor (Infineon TLE493D-W2B6), which was placed above the strip, scanned the surface of the strip and measured the magnetic flux densities of different regions.

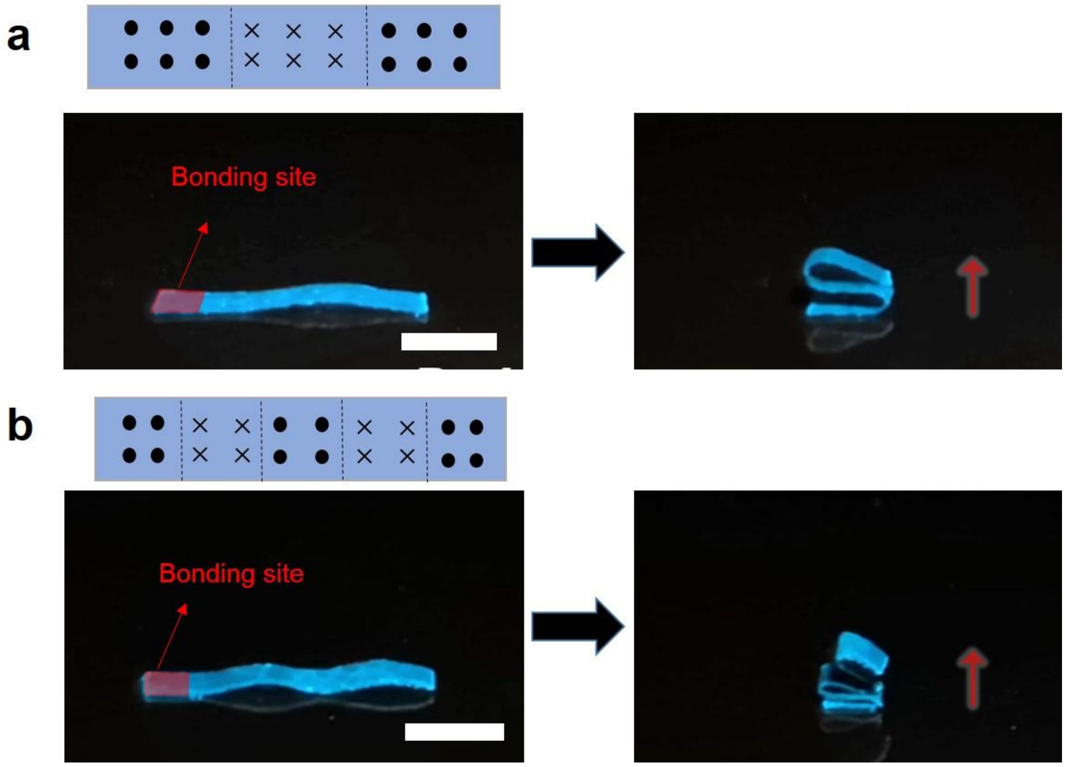

Supplementary Fig. 10. Reprogrammed deformation of a MRSM strip into different 3D configurations. Schematic of two different magnetization patterns in a MRSM strip and their corresponding shape transformation under a magnetic field of 150 mT, which is perpendicular to the films as indicated by the red arrow in images. Since the net magnetization of these patterns is pointing upwards, the actuation results would differ depending on the position where the permanent magnet is placed. To guarantee the stable shape transformation, the leftmost segment of the MRSM film was fixed to the ground by water washable glue. Scale Bars: 5 mm.

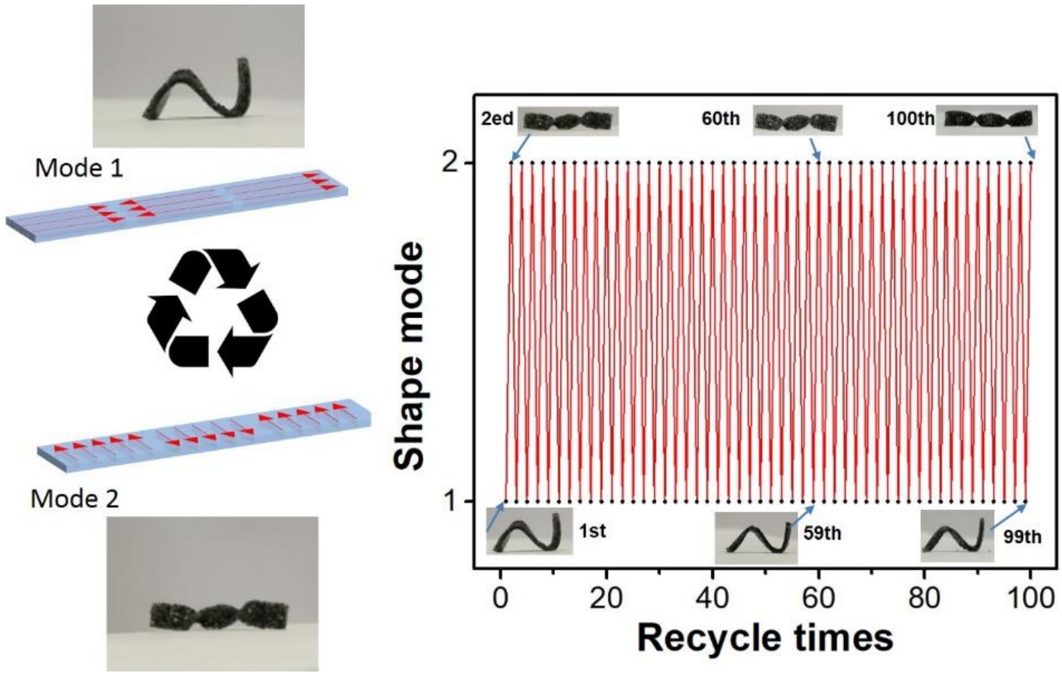

Supplementary Fig. 11. Reversibility and stability of programming the magnetic anisotropies in a MRSM strip. The magnetic anisotropies were alternatively programmed in a MRSM strip, and then the shape morphing was actuated for 100 cycles. During the test, the shape-morphing behaviors were well maintained, suggesting a great reprogramming capability of MRSM.

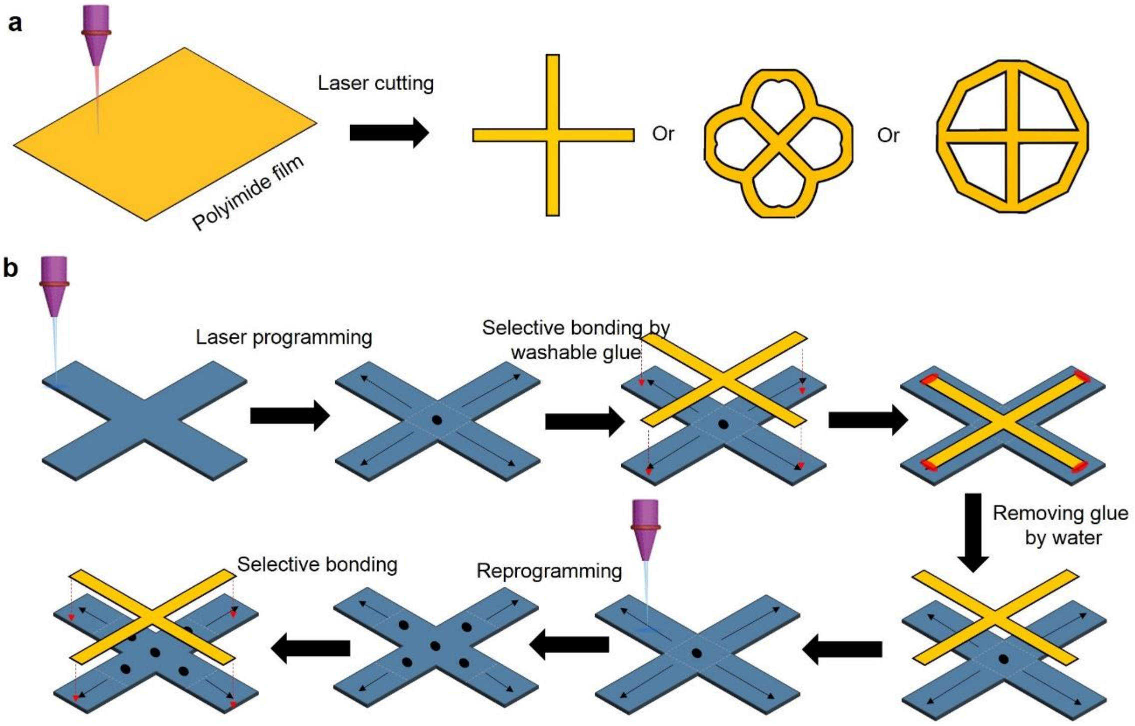

Supplementary Fig. 12. Fabrication steps of MRSM substrates for responsive 3D structure assembly. a, Schematic showing fabrication of a PI kirigami pattern (thickness = 25 μm) by laser cutting. **b,** Schematic showing fabrication of a shape-reprogrammable magnetic buckling device. A water washable glue was used to selectively bond the PI kirigami pattern to the MRSM film. Before recoding a new magnetic pattern in the MRSM film, the PI kirigami pattern was removed from magnetic film by immersed in water.

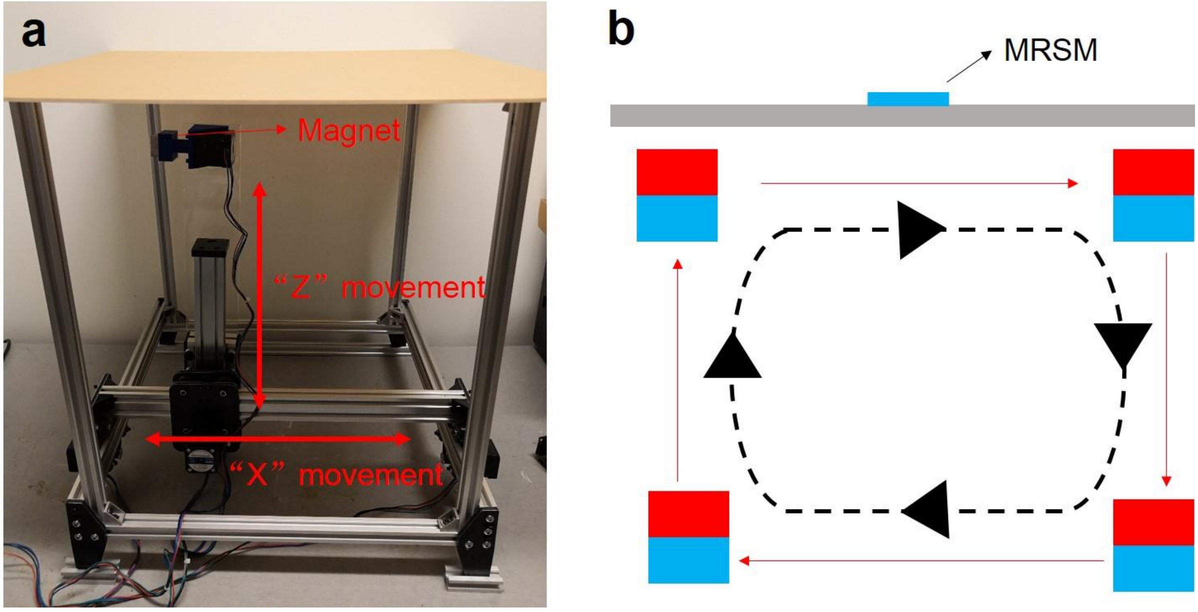

Supplementary Fig. 13. Dynamic magnetic field generation system. a,b, Photograph and schematic showing the system that generates dynamic magnetic field to create the various locomotion modes in a MRSM soft robot. The actuation of the soft robots is driven by a N52 1-inch permanent magnet that is moved by a stepper motor to generate a dynamic magnetic field. The travel distance of the motor is ~ 20 cm.

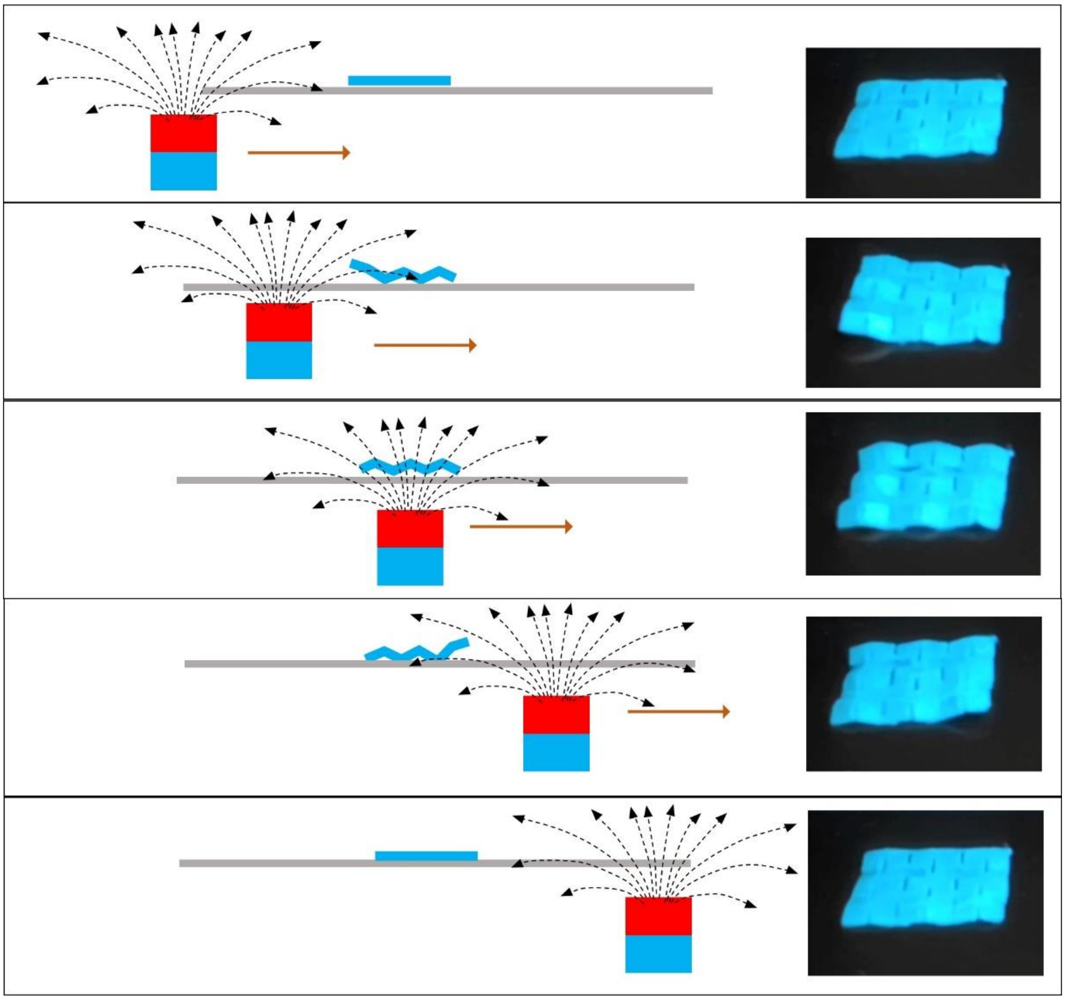

Supplementary Fig. 14. Locomotion process of the earthworm-like soft robot. Schematic showing the locomotion process of a MRSM soft robot under a dynamic magnetic field generated by linear motion of a permanent magnet.

Note: The locomotion mimics the peristalsis of an earthworm. As the magnet moves close to the MRSM soft robot, the magnetic field increases, resulting in contraction motion of its body. As the magnet passes the soft robot, the magnetic field fades, resulting in shape recovery to its original shape. By repeating the above actuation process, the MRSM soft robot repeats this locomotion mode like the peristalsis motion of an earthworm.

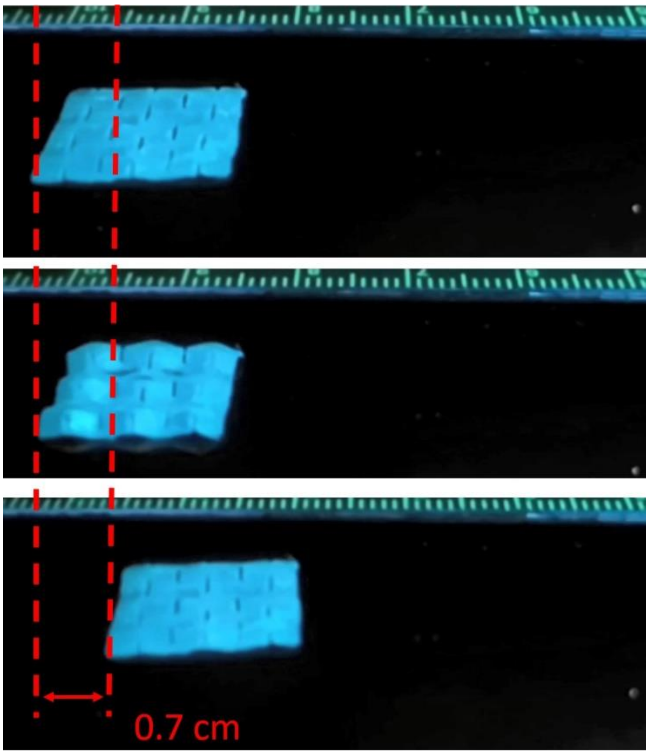

Supplementary Fig. 15. Locomotion ability of the earthworm like soft robot. Photograph showing the moving distance of an earthworm like soft robot after 10 cycles of motion as shown in Supplementary Fig.14.

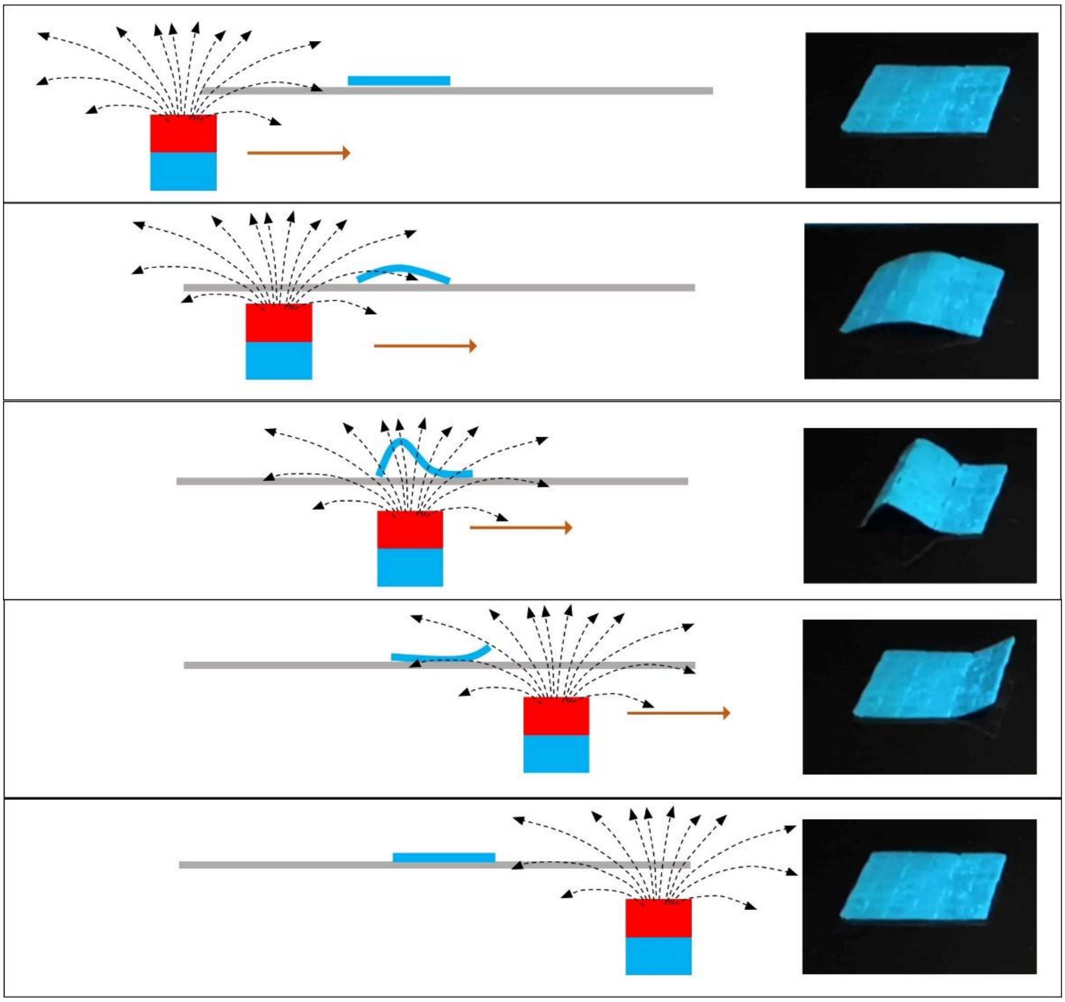

Supplementary Fig. 16. Locomotion process of the inchworm like soft robot. Schematic showing the locomotion process of a MRSM soft robot under a dynamic magnetic field generated by linear motion of a permanent magnet. The moving mimics the crawling motion of an inchworm.

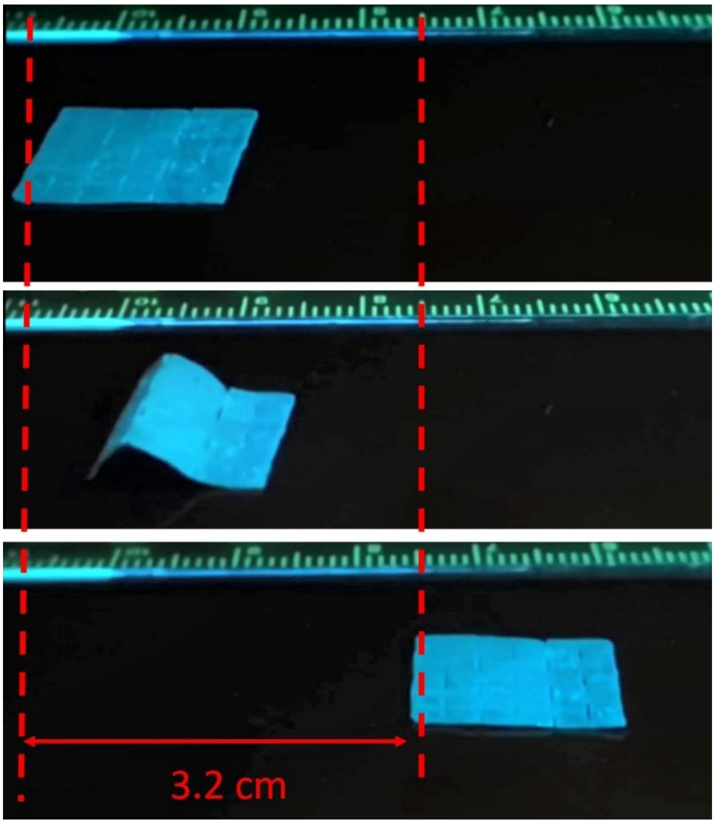

Supplementary Fig. 17. Locomotion ability of the inchworm like soft robot. Photograph showing the moving distance of an inchworm like soft robot after 10 cycles of motion as shown in Supplementary Fig.16.

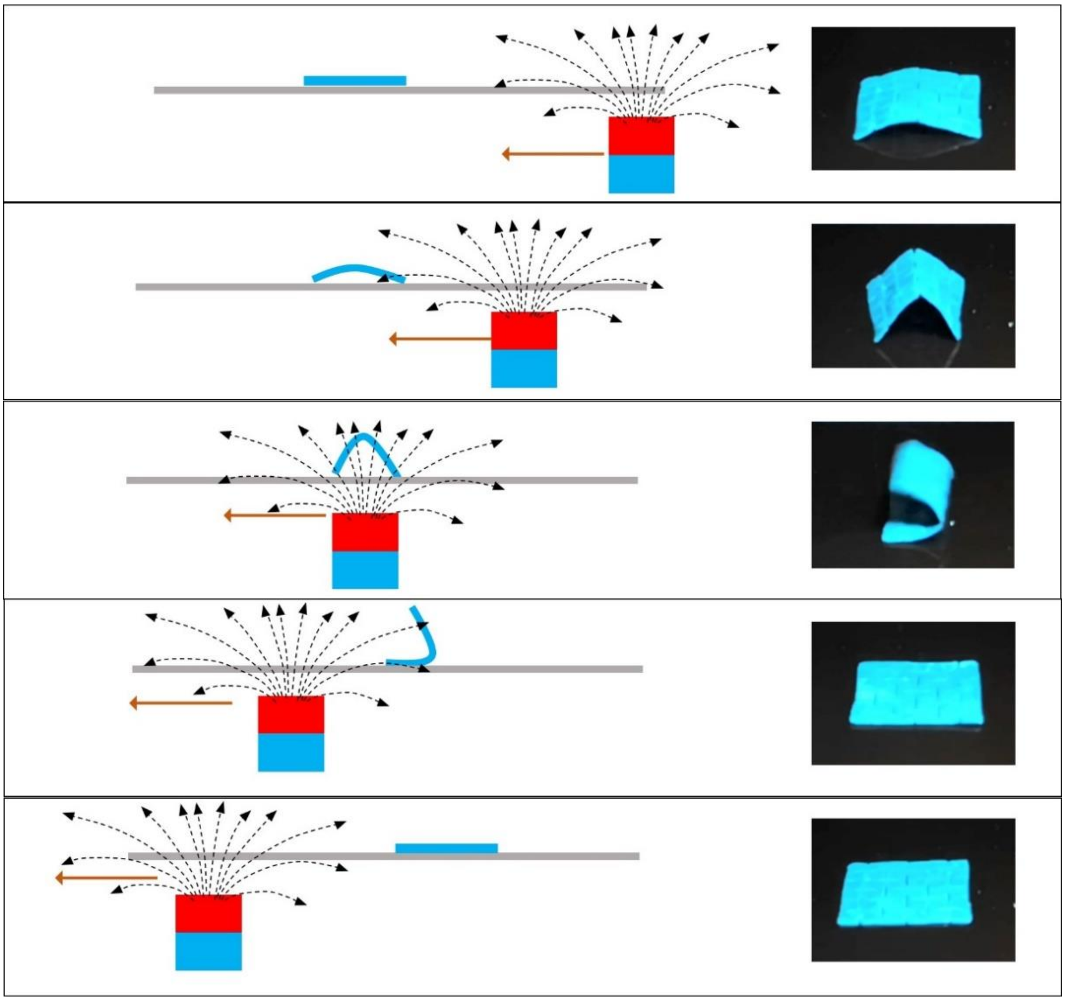

Supplementary Fig. 18. Locomotion process of the pill bug like soft robot. Schematic showing a locomotion process of a MRSM soft robot under a dynamic magnetic field generated by linear motion of a permanent magnet. The moving mimics the rolling motion of a pill bug.

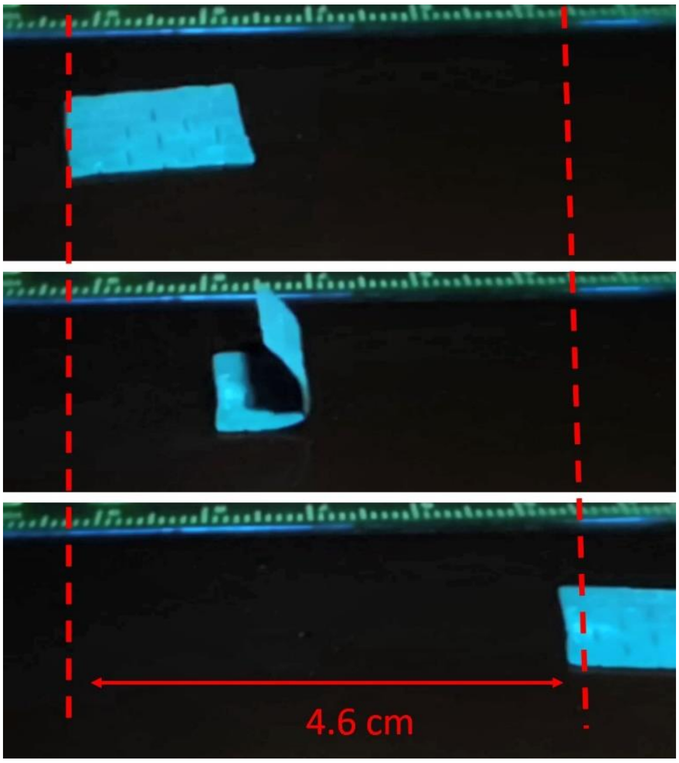

Supplementary Fig. 19. Locomotion ability of the pill bug like soft robot. Photograph showing the moving distance of an inchworm like soft robot after 3 cycles of motion as shown in Supplementary Fig.18.
