# Supplementary material for: Laser reprogramming magnetic anisotropy in soft composites for reconfigurable 3D shaping
Source: Nat Commun. 2020 Dec 10;11:6325. doi: 10.1038/s41467-020-20229-6 (PMC7730436; doi:10.1038/s41467-020-20229-6)
Supplement: Supplementary file 3 — Description of Additional Supplementary Files [file 41467_2020_20229_MOESM3_ESM.pdf]

File Name: Supplementary Movie 1

Description: Reorientation of NdFeB MPs in NdFeB@PCL MPs by an external magnetic field when heated above the melting temperature of PCL.

File Name: Supplementary Movie 2

Description: Different magnetic deformation modes in a MRSM strip.

File Name: Supplementary Movie 3

Description: Different magnetic deformation modes in a square-hollow MRSM film.

File Name: Supplementary Movie 4

Description: Different magnetic deformation modes in a six-armed MRSM film.

File Name: Supplementary Movie 5

Description: Different magnetic deformation modes in a mesh-shaped MRSM film.

File Name: Supplementary Movie 6

Description: Demonstration of MRSM substrates for responsive 3D structure assembly.

File Name: Supplementary Movie 7

Description: Demonstration of MRSM as a multistate electrical switch.

File Name: Supplementary Movie 8

Description: Locomotion process of the earthworm-like magnetic soft robot

File Name: Supplementary Movie 9

Description: Locomotion process of the inchworm-like magnetic soft robot

File Name: Supplementary Movie 10

Description: Locomotion process of the pill bug-like magnetic soft robot
