# Supplementary material for: Laser reprogramming magnetic anisotropy in soft composites for reconfigurable 3D shaping
Source: Nat Commun. 2020 Dec 10;11:6325. doi: 10.1038/s41467-020-20229-6 (PMC7730436; doi:10.1038/s41467-020-20229-6)
Supplement: Supplementary file 2 — Peer Review File [file 41467_2020_20229_MOESM2_ESM.docx]

Reviewers’ Comments

Reviewer #1 (Remarks to the Author):

This work proposed a novel way to fabricate reprogrammable magnetic structures by embedding magnetic materials encapsulated by phase-change medium in polymer matrix. The authors presented a variety of designs as well as their shape transformations and demonstrated the durability of reprogrammable structures. The authors also gave a good review of responsive materials under different stimuli and adequate introduction of magnetic anisotropy encoding strategies. The method is very interesting and is a clever twist on other published related methods.

For the two reasons below, I feel that the work will be of interest to the community due to its novelty, but ultimately may not be solving a real problem in the field and thus unlikely to be adopted widely.

1) One issue with the method is a low magnetization. The authors use a very large field (150 mT) to actuate the devices, which is much higher than that in some of the relevant works. It would be difficult, for example, to apply a dynamic and controlled field of that strength over a distance of more than a few cm. This is perhaps why the authors do not show motions of the soft robots over more than one cycle of motion. The authors should critically compare their effective magnetization strength with the leading literature in this area.

2) Another issue is that the reprogramming requires visual access for the laser, and careful registration to be made. Presumably, the device is removed manually from the workspace and placed back into the fabrication setup to do this (this process is not shown in the paper or videos even though it seems very important). This would preclude the reprogramming when deployed, especially in restricted areas such as in biomedical applications. I am not able to think of a scenario where reprogramming would be useful under this restriction. The authors should say more explicitly how they envision the reprogramming would be used effectively in any target application. These devices are cheap to make in large quantities. Why not just send in a new one if a different bending profile is needed?

Further comments:

To reprogram magnetic anisotropy, one thing that may have effect on the magnetic flux densities of NdFeB@PCL composite is the settling time, which is the time that MPs take to reorient with the external field, in this case it’s also the time interval between PCL’s melting and cooling. So it would be better to indicate that parameter or the sweeping speed of laser.

For the MRSM strip in Figure 2C, the net magnetization is pointing upwards, the actuation results would differ depending on the position where they place the permanent magnet or adhesive conditions of different segments to the ground. It seems the leftmost segment is glued to the ground to generate this ‘S’ shape under actuation field. I expect the authors to clarify the conditions of this shape transformation in the content.

In terms of the functional applications such as buckling assembly, there is cost to recycle the MRSM substrate from the passive film for the reprogramming procedure. For example, splitting two layers at bonding sites will increase time cost and may cause damage to the structure. In this case, I expect the author to state the advantage of reprogramming over the re-fabrication. If we can do the on-board reprogramming, which I cannot tell whether they have already done in this way, that is definitely an efficient way to model reconfigurable structures.

I wonder how position registration is performed for reprogramming? The accuracy of this will determine the reprogrammability.

What is the potential and limits for scaling this to smaller sizes?

The content is well-organized, supplementary documents including figures and videos are abundant and clear to support the arguments. However, there are many typos and syntax errors throughout the paper. Some examples include ‘MMPs’ in Fig.2B, ‘continues’ in line 14 page 6, and the last sentence in page 1.

Reviewer #2 (Remarks to the Author):

The authors proposed a promising method to combine the thermal configuration and magnetic actuation of soft composites to achieve the tunability and reconfiguration of novel structures using direct laser writing for multiple applications. This is a wonderful and innovative work for developing programmable composites.

The composite films are made from elastic matrix and magnetic particles encapsulated by a phase change polymer. Once the phase change polymer was temporary melt by transient laser heating, the orientation of the magnetic particles can be re-aligned upon change of the external magnetic field. This is a smart strategy to make use of the strength of soft polymer (silicone) and PCL.

The paper was well organized and written except some minor grammar errors. The statements are fully supported by the experimental and numerical results. Interesting demonstrations for complex shape change and soft robotics were performed. It can be considered to be published after addressing the following issues listed below:

1. Fig. 1c is a little confusing. Authors should revise it to be concise for clearly delivering the key ideas/steps. It is not clear how the profiles in Fig.1e are formed.

2. Laser-writing is good for a higher resolution patterning, but not as good for a large area fabrication. For the proposed design, usually a large area surface processing will need. The authors should discuss how to get this done easily and efficiently.

3. Fig. 2 C&D the magnetic directions should be pointed out and explained in the captions. For the description of Fig. 2C and 2D, it is not suitable to call them “crease” (page 9) based on the morphology and profiles. More accurate to describe it as “fold” or “bend”.

4. page 15, “in here” should be “herein”. Other expression issues should also be checked and corrected.

5. Fig. 5 and 6 should give a more details captions with self-contained key information for easier following up by readers. In Fig.5B and 5C, the schematics are confusing with the experiment images, no essential information were delivered. It is suggested to remove them from the figure.

6. In the demonstration of the soft robotics, more details on the actuation and operation should be added for easier understanding.

Reviewer #3 (Remarks to the Author):

“Laser Reprogramming Magnetic Anisotropy in Soft Composites for Reconfigurable 3D Shaping” by Deng, Sattari, Xie, Liao, Yan and Lin. The paper describes a composite with magnetic inclusions inside a polymer capsule inside silicone that can be locally heated to re-align the ferromagnetic particles and thereby define the local magnetization. The aligned hard magnetic powders cool after illumination and the magnetization direction is preserved. Patterns of magnetization can then be used to actuate the soft shape.

Much of the paper appears like a combination of well-known ideas. The real novelty could be the claim of reconfigurability. This is achieved by overwriting the same shape in the direct laser writing setup. Technically, the authors have achieved reconfigurability, but it is not practical. Direct laser writing is slow. The shape must be processed twice: First, when making it, then when it is reconfigured. Well, one might as well make a new structure in the same time. Therefore, I do not find the solution particularly original or useful. I do not know what problem the method can help solve.

Since direct laser writing of shapes with magnetic inclusions is not new (e.g. https://doi.org/10.1063/1.2954011 , DOI 10.1007/s10544-013-9791-7) and since the idea of Reprogrammable Ferromagnetic Domains for Reconfigurable SoftMagnetic Actuators (https://doi.org/10.1021/acs.nanolett.0c01418) is also not new, the only real novelty is the use of direct laser writing to achieve reprogrammable structures. However, I do not find that this is particularly surprising or that it is useful.

The reviewers brought up some technique questions or provided suggestions to further strengthen our work. The “Responses to the referee’s comments” is attached below. To make it clear, we have used *italic* fonts for the reviewers’ comments, **bold** fonts for our replies and blue fonts for our revisions in the manuscript. The revised portion has been highlighted in red in our revised manuscript.

Reviewers' comments:

Reviewer #1 (Remarks to the Author):

*This work proposed a novel way to fabricate reprogrammable magnetic structures by embedding magnetic materials encapsulated by phase-change medium in polymer matrix. The authors presented a variety of designs as well as their shape transformations and demonstrated the durability of reprogrammable structures. The authors also gave a good review of responsive materials under different stimuli and adequate introduction of magnetic anisotropy encoding strategies. The method is very interesting and is a clever twist on other published related methods.*

*For the two reasons below, I feel that the work will be of interest to the community due to its novelty, but ultimately may not be solving a real problem in the field and thus unlikely to be adopted widely.*

**Response: We appreciate reviewer’s acknowledgement on the novelty of our work. The point-to-point responses according to the reviewer’s constructive suggestions have been addressed in the revised manuscript.**

*(1) One issue with the method is a low magnetization. The authors use a very large field (150 mT) to actuate the devices, which is much higher than that in some of the relevant works. It would be difficult, for example, to apply a dynamic and controlled field of that strength over a distance of more than a few cm. This is perhaps why the authors do not show motions of the soft robots over more than one cycle of motion. The authors should critically compare their effective magnetization strength with the leading literature in this area.*

**Response: Thanks to reviewer for bringing up such a great question. The reason of the relatively low magnetization is because of the low pre-magnetization of the NdFeB microparticles (MPs). In our work, we adopted a very simple pre-magnetizing method by assembling two N52 1-inch permanent magnets to generate a uniform magnetic field. This method is similar to the one shown in a previous literature (Sci. Robot. 2019, 4(29): eaav4494.). This paper shows that the fabricated magnetic films have magnetization of 1-3 mT. Our samples show comparable magnetization of ~ 1.2 mT. To lower the actuation magnetic field, a much larger pre-magnetization field is needed. Unfortunately, we do not have such a facility. As an alternative solution in our work, which was also employed in other publication (e.g. Nature 2018, 558(7709): 274-279), an actuation magnetic field of 150 mT was utilized by applying a cheap N52 1-inch permanent magnet (costing only eight dollars). It is strong enough to actuate the fabricated soft robots to move several centimeters in each cycle. As the magnet is dynamically controlled by the moving stage, the soft robots show varied moving distance but very consistent motion behaviors in each actuation cycle (see supplementary videos, Figure S).**

**Nevertheless, the science or novelty of our work is that the laser programming the magnetic anisotropy of the MRSM films can achieve reconfigurable 3D shaping. The laser induced reorientation of NdFeB MPs yields a magnetic flux density of ~ 90% of the saturated magnetic flux density in the MRSM films. It is larger than the one shown in a previous literature (Nature 2018, 558(7709): 274-279), in which a nozzle-based 3D printer was used to only achieve 63%–64% of the saturated magnetic flux density.**

**Correspondingly we added the following discussion in the manuscript:**

**We added** “The NdFeB MPs have an average size of 5 μm (Figure S4), which were pre-magnetized in a 1.1 T uniform magnetic field formed by two N52 1-inch permanent magnets with a 3-mm apart.^26^” **in Page 7.**

**We added** “The magnetization of the MRSM film reaches to ~ 1.2 mT, which is compared to the fabricated samples shown in a previous study.^26^ The laser induced reorientation of NdFeB MPs yields a magnetic flux density of ~ 90% of the saturated magnetic flux density in the MRSM films, which is much higher than those realized by a 3D printing method.^24^” **in Page 8.**

Reference:

24. Kim Y, Yuk H, Zhao R, Chester SA, Zhao X. Printing ferromagnetic domains for untethered fast-transforming soft materials. *Nature* 2018, **558**(7709)**:** 274-279.

26. Xu T, Zhang J, Salehizadeh M, Onaizah O, Diller E. Millimeter-scale flexible robots with programmable three-dimensional magnetization and motions. *Sci. Robot.* 2019, **4**(29)**:** eaav4494.

**Correspondingly we added the following description in the manuscript:**

**We added** “By repeating the actuation process, the MRSM soft robot repeats this locomotion mode like the peristalsis of an earthworm, which moves 0.7 cm after 10 cycles of motion. (Figure 6A, Figure S15 and Movie S8).” **in Page 18.**

**We added** “By repeating the actuation process, the MRSM soft robot moves forward by cycling movement of arching and stretching just like a creeping inchworm, which moves 3.2 cm after 10 cycles of motion. (Figure 6B, Figure S17 and Movie S9).” **in Page 18.**

**We added** “by using the same actuation process (Figure S17), we can expect a rolling locomotion of the MRSM soft robot as a pill bug does, which moves 4.6 cm after 3 cycles of motion. (Figure 6C, Figure S19 and Movie S10).” **in Page 18.**

**We added the following figure in Supporting Information as Figure S15:**

**Figure S15**. Photograph showing the moving distance of an earthworm like soft robot after 10 cycles of motion as shown in Figure S14.

**We added the following figure as Figure S17 in Supporting Information:**

**Figure S17**. Photograph showing the moving distance of an inchworm like soft robot after 10 cycles of motion as shown in Figure S16.

**We added the following figure in Supporting Information as Figure S19:**

**Figure S19**. Photograph showing the moving distance of an inchworm like soft robot after 3 cycles of motion as shown in Figure S18.

*(2) Another issue is that the reprogramming requires visual access for the laser, and careful registration to be made. Presumably, the device is removed manually from the workspace and placed back into the fabrication setup to do this (this process is not shown in the paper or videos even though it seems very important). This would preclude the reprogramming when deployed, especially in restricted areas such as in biomedical applications. I am not able to think of a scenario where reprogramming would be useful under this restriction. The authors should say more explicitly how they envision the reprogramming would be used effectively in any target application. These devices are cheap to make in large quantities. Why not just send in a new one if a different bending profile is needed?*

**Response: This is a great question. To program the magnetic anisotropy, it is necessary to align the MRSM film to the laser scanning pathway. To make the position registration, we adopt a simple method as shown in Figure S2B. A polyimide (PI) tape was placed on the DLW platform, then the laser scanned the PI surface to induce laser induced graphene (LIG). As the laser scanning pathway is the same to the one used for programming the magnetic anisotropy, the LIG can be used as the marker. Thus, it avoids additional marker for visual access.**

**Correspondingly we added the following description in the manuscript:** “A customized DLW system to achieve such a goal is shown in Figure 1B. Details of the system design and construction are described in Figure S2. To align the MRSM film to the laser scanning pathway, laser induced graphene^32^ was formed on the polyimide surface as a marker (Figure S2B), which enables to position registration without visual access to the laser**.**” **in Page 5.**

**We added the following figure in supporting information as Figure S2B:**

**Figure S2**. (B) Schematic showing the process of position registration. A polyimide (PI) tape is placed on the DLW platform. A polyimide (PI) tape was placed on the DLW platform, then a blue laser (0.1 W) scanned the PI surface to induce laser induced graphene (LIG). As the laser scanning pathway is the same to the one used for programming the magnetic anisotropy, the LIG can be used as the marker for position registration. Finally, a glass slide is covered on the sample to fix the position of sample. After that, the sample is ready for laser programming.

**We agree with the reviewer that it will be a very long way to achieve the practical application of the proposed reprogrammable magnetic films. However, we believe that the concept of reprogrammable materials is an intriguing topic in material science. It would lay new foundation to soft robots that can adapt the changing environment through reconfigurable shape morphing. As the reviewer indicates, the challenge would be how to do in situ reprogramming via direct laser writing. Honestly speaking, it would be a very challenging task to achieve, which is beyond the scope of the current work. We envision that as the technology evolves with the continuous effort, new magnetic and laser control platforms would be developed to make the practical applications possible.**

*(3) To reprogram magnetic anisotropy, one thing that may have effect on the magnetic flux densities of NdFeB@PCL composite is the settling time, which is the time that MPs take to reorient with the external field, in this case it’s also the time interval between PCL’s melting and cooling. So it would be better to indicate that parameter or the sweeping speed of laser.*

**Response: Reviewer made a great point. However, it is almost impossible to measure the exact time interval between melting and cooling of the PCL, as the interval may be largely influenced by many factors. For instance, the sweeping speed of the laser is one of them, as indicated in our revised manuscript. The DLW generates transient heat on the exposed regions. The transient heat can effectively limit thermal diffusion to non-exposed regions, thus increasing the patterning resolution. In addition, rapid cooling enables quick fixation of the magnetization profiles in the exposed regions, thus avoiding interference when the direction of the programming magnetic field is changing during the continuous laser scanning process (Figure S2C).**

**Correspondingly we added the following description in the manuscript: “**The Adurino board controls the pathway of a laser head (speed = 200 mm/min) and also the direction of the magnetic field from a cube permanent magnet.**” In the caption of Figure S2.**

*(4) For the MRSM strip in Figure 2C, the net magnetization is pointing upwards, the actuation results would differ depending on the position where they place the permanent magnet or adhesive conditions of different segments to the ground. It seems the leftmost segment is glued to the ground to generate this ‘S’ shape under actuation field. I expect the authors to clarify the conditions of this shape transformation in the content.*

**Response: Thanks to Reviewer for bringing up such a great comment. As indicated, the actuation results in Figure 2C and Figure 2D would differ depending on the position where the permanent magnet is placed. To solve this problem, we use a washable glue to attach the leftmost segment of the MRSM film to the ground.**

**Correspondingly we added the following description in the manuscript:** “It should be noted that the leftmost segment of magnetic film was fixed to the ground by water washable glue to guarantee the stable shape transformations (Figure S10).” **in Page 10.**

**We added the following figure in supporting information as Figure S10:**

**Figure S10.** Schematic of two different magnetization patterns in a MRSM strip and their corresponding shape transformation under a magnetic field of 150 mT, which is perpendicular to the films as indicated by the red arrow in images. Since the net magnetization of these patterns is pointing upwards, the actuation results would differ depending on the position where the permanent magnet is placed. To guarantee the stable shape transformation, the leftmost segment of the MRSM film was fixed to the ground by water washable glue. Scale Bars: 5 mm.

*(5) In terms of the functional applications such as buckling assembly, there is cost to recycle the MRSM substrate from the passive film for the reprogramming procedure. For example, splitting two layers at bonding sites will increase time cost and may cause damage to the structure. In this case, I expect the author to state the advantage of reprogramming over the re-fabrication. If we can do the on-board reprogramming, which I cannot tell whether they have already done in this way, that is definitely an efficient way to model reconfigurable structures.*

**Response: We thank reviewer for making such great suggestions. It is quite intriguing recently to endow functional materials shape reprogrammable capability, despite that it is still far from practical applications. Nevertheless, reviewer inspired us to take a closer look at the fabrication process and structures fabrication. Thus, more experimental details of buckling assembly were added in our revised manuscript.**

**Correspondingly we change “**Figure 4A is the schematic showing the fabrication steps**”**

**To** **“**The fabrication steps are shown in Figure 4A and Figure S12.**” in Page 14.**

**We added the following figure in supporting information as Figure S12:**

**Figure S12.** (A) Schematic showing fabrication of a PI kirigami pattern (thickness = 25 μm) by laser cutting. (B) Schematic showing fabrication of a shape-reprogrammable magnetic buckling device. A water washable glue was used to selectively bond the PI kirigami pattern to the MRSM film. Before recoding a new magnetic pattern in the MRSM film, the PI kirigami pattern was removed from magnetic film by immersed in water.

*(6) I wonder how position registration is performed for reprogramming?* *The accuracy of this will determine the reprogrammability.*

**Response: We thank reviewer for bringing up the questions about the experimental details. To make the position registration, we adopt a simple method as shown in Figure S2B. A polyimide (PI) tape was placed on the DLW platform, then the laser scanned the PI surface to induce laser induced graphene (LIG). As the laser scanning pathway is the same to the one used for programming the magnetic anisotropy, the LIG can be used as the marker. Thus, it avoids additional marker for visual access.**

**From our final results, such DLW technique is efficient to program magnetic anisotropies, in which the magnetic anisotropies are precisely encoded in the magnetic final as we expected.**

**Correspondingly we added the following description in the manuscript:**

“A customized DLW system to achieve such a goal is shown in Figure 1B. Details of the system design and construction are described in Figure S2. To align the magnetic film to the pathway of laser writing, laser induced graphene^32^ was formed on platform as marker (Figure S2B), which enable us for position registration without visual access for the laser**.**”**in Page 5.**

*(7) What is the potential and limits for scaling this to smaller sizes?*

**Response: We thank reviewer for bringing this great question. The size scale is limited by the resolution of our laser. As described in Method section, we used a CO_2_ laser to cut samples into desired geometries and used a blue laser to program magnetic anisotropy inside the samples. However, the resolutions of our lasers are in the scale of ~ 200 µm. With those, a millimeter-scale four-armed soft robot was fabricated (Figure S3). The size of each arm is 1.25 mm x 500 μm. A magnetization profiles as shown in Figure S3A was encoded in the MRSM film which was actuated into a soft robot under a magnetic field. We believe that the micro-scale structures will be rendered if a higher resolution laser can be employed. Such a possibility is under investigation and will be reported in a follow-up work.**

**Correspondingly we added the following description in the manuscript:**

“Such position registration by using LIG as the marker guarantees the accuracy of the laser programming. The patterning resolution is estimated to be ~ 300 μm, rendering a soft robot in a millimeter scale (Figure S3).” **in Page 5**.

**We added the following figure in supporting information:**

Figure S3. (A) Schematic of a millimeter-scale four-armed soft robot. The size of each arm is 1.25 mm x 500 μm. (B) Shape transformation of a four-armed soft robot under an actuation magnetic field of 150 mT, which is perpendicular to the films as indicated by the red arrow.

*(8) The content is well-organized, supplementary documents including figures and videos are abundant and clear to support the arguments. However, there are many typos and syntax errors throughout the paper. Some examples include ‘MMPs’ in Fig.2B, ‘continues’ in line 14 page 6, and the last sentence in page 1.*

**Response: Thanks for reviewer’s good suggestion. We examine the manuscript again. There are actually several typos and grammar mistakes. We have changed these mistakes in our revised manuscript. The revisions are highlighted in red in the manuscript.**

Reviewer #2 (Remarks to the Author):

*The authors proposed a promising method to combine the thermal configuration and magnetic actuation of soft composites to achieve the tunability and reconfiguration of novel structures using direct laser writing for multiple applications. This is a wonderful and innovative work for developing programmable composites.*

*The composite films are made from elastic matrix and magnetic particles encapsulated by a phase change polymer. Once the phase change polymer was temporary melt by transient laser heating, the orientation of the magnetic particles can be re-aligned upon change of the external magnetic field. This is a smart strategy to make use of the strength of soft polymer (silicone) and PCL.*

*The paper was well organized and written except some minor grammar errors. The statements are fully supported by the experimental and numerical results. Interesting demonstrations for complex shape change and soft robotics were performed. It can be considered to be published after addressing the following issues listed below:*

**We appreciate your positive comments. The point-to-point responses according to your constructive suggestions have been addressed in the revised manuscript.**

*(1). Fig. 1c is a little confusing. Authors should revise it to be concise for clearly delivering the key ideas/steps. It is not clear how the profiles in Fig.1e are formed.*

**Thanks for reviewer’s good suggestion. Figure 1C-E were updated as shown in the following.**

*(2). Laser-writing is good for a higher resolution patterning, but not as good for a large area fabrication. For the proposed design, usually a large area surface processing will need. The authors should discuss how to get this done easily and efficiently.*

**Response: We appreciate the reviewer’s good suggestion. The patterning area surface is limited by the scanning dimensions of the laser system. In our system, a total area of 20cm*20cm can be realized. To make a larger patterning area, the dimension of the DLW system must be enlarged. To increase the scanning more efficiency, the point-to-point laser can be change to a flex exposure laser. Together with a digital projector, irradiation of a larger area can be achieved at one time.**

*(3). Fig. 2 C&D the magnetic directions should be pointed out and explained in the captions. For the description of Fig. 2C and 2D, it is not suitable to call them “crease” (page 9) based on the morphology and profiles. More accurate to describe it as “fold” or “bend”.*

**Response: Thanks to reviewer’s good suggestion. We have followed reviewer’s advice to add the description about the magnetic directions in captions of the figures. The “crease” indicates the boundaries across the adjacent patterns with opposite magnetic directions. We take the reviewer’s suggestion and name them as “folding creases”.**

**Correspondingly, we added the following description: “**The strip is actuated under a magnetic field of 150 mT, which is perpendicular to the strip as indicated by the red arrow.” **in the caption of Figure 2.**

**We added the following description: “**The magnetic films are actuated under a magnetic field of 150 mT, which is perpendicular to the films as indicated by the red arrow.” **in the caption of Figure 3.**

**We added the following description:** “The magnetic switch is actuated by a magnetic field of 150 mT, which is perpendicular to the device as indicated by the red arrow.” **in the caption of Figure 5.**

**Correspondingly we change “**Meanwhile, in this magnetization pattern, the boundaries across the adjacent patterns form the creases for an origami pattern. Along these creases….**”**

**To** **“**Meanwhile, in this magnetization pattern, the boundaries across the adjacent patterns form the folding creases for an origami pattern. Along these folding creases….**” in Page 10.**

*(4). page 15, “in here” should be “herein”. Other expression issues should also be checked and corrected.*

**Response: Thanks for reviewer’s careful checking. We further polished the language of the manuscript and corrected typos and grammar mistakes in addition to the ones suggested by the reviewer.** **The revisions are highlighted in red in the revised manuscript.**

*(5). Fig. 5 and 6 should give a more details captions with self-contained key information for easier following up by readers. In Fig.5B and 5C, the schematics are confusing with the experiment images, no essential information were delivered. It is suggested to remove them from the figure.*

**Response: Figure 5B and Figure 5c are used to clarify the experimental details about the magnetic switch.**

*(6). In the demonstration of the soft robotics, more details on the actuation and operation should be added for easier understanding.*

**Response: Reviewer made great suggestions.** **The actuation of the soft robots is driven by a N52 1-inch permanent magnet that is moved a stepper motor to generate a dynamic magnetic field. The traveling distance of the motor is ~ 20 cm. The soft robots may travel up to 20 cm. The earthworm like soft robots can move 0.7 cm distance every 10 cycles of motions. The inchworm like soft robot can move 3.2 cm every 10 cycles of motions. And the pill bug like soft robot can move 4.6 cm distance every 3 cycles of motions.**

**Correspondingly we added following information in the manuscript.**

**We added “**The actuation of the soft robots is driven by a N52 1-inch permanent magnet that is moved by a stepper motor to generate a dynamic magnetic field. The travel distance of the motor is ~ 20 cm.” **in the caption of Figure S13**.

**Correspondingly we added the following description in the manuscript:**

**We added** “By repeating the actuation process, the MRSM soft robot repeats this locomotion mode like the peristalsis of an earthworm, which moves 0.7 cm after 10 cycles of motion. (Figure 6A, Figure S14 and Movie S8).” **in Page 18.**

**We added** “By repeating the actuation process, the MRSM soft robot moves forward by cycling movement of arching and stretching just like a creeping inchworm, which moves 3.2 cm after 10 cycles of motion. (Figure 6B, Figure S16 and Movie S9).” **Page 18.**

**We added** “by using the same actuation process (Figure S17), we can expect a rolling locomotion of the MRSM soft robot as a pill bug does, which moves 4.6 cm after 3 cycles of motion. (Figure 6C, Figure S18 and Movie S10).” **in Page 18.**

**We added Figure S15 in Supporting Information:**

**Figure S15**. Photograph showing the moving distance of earthworm like soft robot after 10 cycles of motion as shown in Figure S14.

**We added the following figure in supporting information as Figure S17:**

Figure S17. Photograph showing the moving distance of inchworm like soft robot after 10 cycles of motion as shown in Figure S16.

**We added the following figure in supporting information as Figure S18:**

Figure S19. Photograph showing the moving distance of inchworm like soft robot after 3 cycles of motion as shown in Figure S18.

Reviewer #3 (Remarks to the Author):

*“Laser Reprogramming Magnetic Anisotropy in Soft Composites for Reconfigurable 3D Shaping” by Deng, Sattari, Xie, Liao, Yan and Lin. The paper describes a composite with magnetic inclusions inside a polymer capsule inside silicone that can be locally heated to re-align the ferromagnetic particles and thereby define the local magnetization. The aligned hard magnetic powders cool after illumination and the magnetization direction is preserved. Patterns of magnetization can then be used to actuate the soft shape.*

*Much of the paper appears like a combination of well-known ideas. The real novelty could be the claim of reconfigurability. This is achieved by overwriting the same shape in the direct laser writing setup. Technically, the authors have achieved reconfigurability, but it is not practical. Direct laser writing is slow. The shape must be processed twice: First, when making it, then when it is reconfigured. Well, one might as well make a new structure in the same time. Therefore, I do not find the solution particularly original or useful. I do not know what problem the method can help solve.*

*Since direct laser writing of shapes with magnetic inclusions is not new (e.g. https://doi.org/10.1063/1.2954011 , DOI 10.1007/s10544-013-9791-7) and since the idea of Reprogrammable Ferromagnetic Domains for Reconfigurable SoftMagnetic Actuators (https://doi.org/10.1021/acs.nanolett.0c01418) is also not new, the only real novelty is the use of direct laser writing to achieve reprogrammable structures. However, I do not find that this is particularly surprising or that it is useful.*

**Response: We thank the reviewer for the comments. Reviewer’s reservation on the novelty is based on that the concept of our work is “a combination of well-known ideas”. We respectfully disagree with the conclusion. Getting inspirations from previous works but proposing a new idea can sometimes lead to some important technical advancement. For instance, in this work, we offered a novel methodology to program and reprogram magnetic anisotropies in responsive self-morphing materials by the direct laser writing. We believe that this novel route will inspire more study and move the field forward.**

**The reviewer also mentions that “Direct laser writing is slow”. DLW has been widely used as a scalable surface patterning and cutting tool in manufacturing. The patterning area surface is limited by the scanning dimensions of the laser system. In our system, a total area of 20cm*20cm can be realized. To make a larger patterning area, the dimension of the DLW system must be simply enlarged. To increase the scanning more efficiency, the point-to-point laser can be change to a flex exposure laser. Together with a digital projector, irradiation of a larger area can be achieved at one time.**

**The reviewer also mentioned “The shape must be processed twice: First, when making it, then when it is reconfigured. Well, one might as well make a new structure in the same time.” This concern was also raised by Reviewer #1. We agree with the reviewers that it will be a very long way to achieve the practical application of the proposed reprogrammable magnetic films. However, we believe that the concept of reprogrammable materials is an intriguing topic in material science. It would lay new foundation to soft robots that can adapt the changing environment through reconfigurable shape morphing. As the reviewer indicates, the challenge would be how to do insitu reprogramming via direct laser writing. Honestly speaking, it would be a very challenging task to achieve, which is beyond the scope of the current work. We envision that as the technology evolves with the continuous effort, new magnetic and laser control platforms would be developed to make the practical applications possible.**

**Finally, the reviewer also mentioned “direct laser writing of shapes with magnetic inclusions is not new (e.g. https://doi.org/10.1063/1.2954011, DOI 10.1007/s10544-013-9791-7) and since the idea of Reprogrammable Ferromagnetic Domains for Reconfigurable Soft Magnetic Actuators (https://doi.org/10.1021/acs.nanolett.0c01418) is also not new”, and concluded there is no novelty for our work.**

**We respectfully disagree with the reviewer’s conclusion. Our work is totally different form these literatures. The literature (https://doi.org/10.1063/1.2954011 and DOI 10.1007/s10544-013-9791-7) used the laser irradiation to crosslink magnetic particles and polymer network composite. While our work adopts a photothermal effect of laser to temporarily melt the phase change polymers that encapsulate the magnetic particles, thus enabling the reorientation of magnetic particles under a programming magnetic field. In the other literature (https://doi.org/10.1021/acs.nanolett.0c01418), manual deformation of the magnetic films followed by the magnetization was employed to program the shape change. However, our work utilizes direct laser writing, which is a digitally and precisely controlled patterning tool, to pattern magnetic anisotropy with high design freedom and** [**controllability**](http://www.youdao.com/w/controllability/#keyfrom=E2Ctranslation) **in a soft magnetic composite.**

Reviewers’ Comments

Reviewer #1 (Remarks to the Author):

My technical concerns have been addressed. The authors avoided addressing my question regarding what applications this reprogramming would be useful for, given the strong constraint of line-of-sight access being required. Indeed this is a difficult question, and one which I suggest the authors continue to consider.

Reviewer #2 (Remarks to the Author):

The authors have addressed the reviewers' comments/concerns well. I have no further comment on it. The manuscript is acceptable at its current state.

The “Responses to the referee’s comments” is attached below. To make it clear, we have used *italic* fonts for the reviewers’ comments, **bold** fonts for our replies and blue fonts for our revisions in the manuscript. The revised portion has been highlighted in red in our revised manuscript.

Reviewers' comments:

Reviewer #1 (Remarks to the Author):

*My technical concerns have been addressed. The authors avoided addressing my question regarding what applications this reprogramming would be useful for, given the strong constraint of line-of-sight access being required. Indeed this is a difficult question, and one which I suggest the authors continue to consider.*

**Response: We thank the reviewer for the favorable comment. We agree with the reviewer that it is difficult to achieve the real application of this reprogrammable magnetic film at the current stage. However, reprogrammable and responsive materials have been and will be always an interest in the society of material science. Despite recent progress, it is very difficult to realize practical applications for these materials in the current stage. From both economic and environmental aspects, it is very important to endow functional materials recyclability. The demonstrated reconfigurable polymeric composites show such a characteristics.**

**We added “**Moreover, the reprogrammability of the MRSM films will broaden the potential of responsive materials in various fields from both economic and environmental aspects.” **in Line 18 of Page 12.**

Reviewer #2 (Remarks to the Author):

*The authors have addressed the reviewers' comments/concerns well. I have no further comment on it. The manuscript is acceptable at its current state.*

**Response: We thank the reviewer for accepting the manuscript for publication.**
